# Harnessing Competitive Interactions to Regulate Supramolecular “Micelle-Droplet-Fiber” Transition and Reversibility in Water

**DOI:** 10.1021/jacs.4c11285

**Published:** 2024-10-15

**Authors:** Heleen Duijs, Mohit Kumar, Shikha Dhiman, Lu Su

**Affiliations:** † Division of Biotherapeutics, Leiden Academic Centre for Drug Research (LACDR), Leiden University, Einsteinweg 55, 2333 CC Leiden, The Netherlands; ‡ Department of Chemistry, Johannes Gutenberg University in Mainz, Duesbergweg 10-14, D-55128 Mainz, Germany

## Abstract

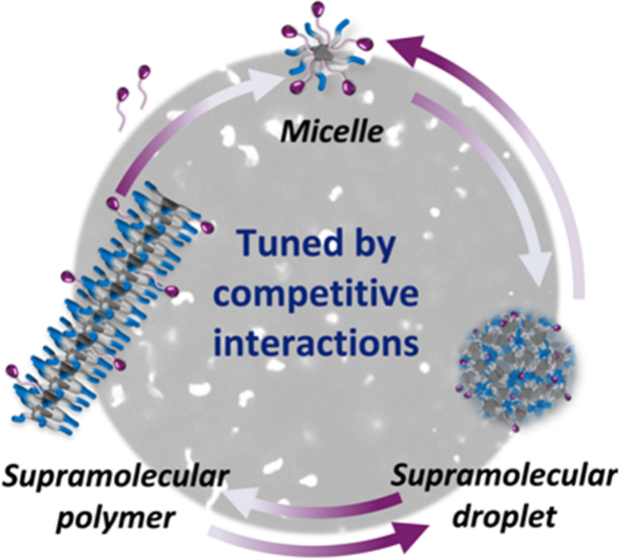

The supramolecular assembly of proteins into irreversible fibrils is often associated with diseases in which aberrant phase transitions occur. Due to the complexity of biological systems and their surrounding environments, the mechanism underlying phase separation-mediated supramolecular assembly is poorly understood, making the reversal of so-called irreversible fibrillization a significant challenge. Therefore, it is crucial to develop simple model systems that provide insights into the mechanistic process of monomers to phase-separated droplets and ordered supramolecular assemblies. Such models can help in investigating strategies to either reverse or modulate these states. Herein, we present a simple synthetic model system composed of three components, including a benzene-1,3,5-tricarboxamide-based supramolecular monomer, a surfactant, and water, to mimic the condensate pathway observed in biological systems. This highly dynamic system can undergo “micelle-droplet-fiber” transition over time and space with a concentration gradient field, regulated by competitive interactions. Importantly, manipulating these competitive interactions through guest molecules, temperature changes, and cosolvents can reverse ordered fibers into a disordered liquid or micellar state. Our model system provides new insights into the critical balance between various interactions among the three components that determine the pathway and reversibility of the process. Extending this “competitive interaction” approach from a simple model system to complex macromolecules, e.g., proteins, could open new avenues for biomedical applications, such as condensate-modifying therapeutics.

## Introduction

Supramolecular assembly is fundamental to the formation of many complex biological structures, for instance, protein filaments, DNA, and membranes.^1^ Its noncovalent nature enables dynamic assemblies of divergent building blocks, allowing for drastic conformational or functional changes in response to slight alterations in the chemical environment.^2−5^ Competitive interactions are critical in modulating the chemical environment, thereby influencing the supramolecular energy landscape and the resulting order in the structures.^1^ Among these, the supramolecular assembly of proteins into fibrils can be mediated by the process of liquid–liquid phase separation (LLPS).^7,8^ This process clusters monomers into solute-rich droplets, thereby facilitating nucleation and promoting fiber formation.^9^ This LLPS-mediated fiber growth is displayed by natural building blocks, such as intrinsically disordered proteins or peptides.^8,10−14^ The LLPS and self-assembly can be a multistep desolvation process driven by the competitive binding of solvents to peptides.^15^ Moreover, it has been observed that even small synthetic molecules can form liquid droplets, either at equilibrium or at the metastable state *enroute* to supramolecular polymerization.^7,8,16−18^ This suggests a general mechanism of LLPS-mediated supramolecular polymerization, proving the versatility of synthetic supramolecular building blocks as mimics of natural systems.^19^ This emphasizes the role of phase separation in driving supramolecular polymerization, which is yet to be completely understood.

In the biological realm, the aberrant transition from liquid droplets into ordered solid protein aggregates is increasingly associated with the onset and progression of diseases.^20−28^ Novel mechanistic insights are gradually being uncovered. For example, the RNA concentration affects the phase separation-mediated fibrilization of RNA-binding proteins, demonstrating that competitive interactions can regulate the condensation pathway.^29^ However, the study of reversing protein aggregation remains elusive.^30^ Understanding phase separation-mediated fibrilization should therefore be expanded into a general concept using a simple supramolecular model system. This model system will allow exploration of strategies to intervene with the solid aggregates and return to the liquid state, aiming to develop effective treatments for protein condensate-related diseases.^31^

**Figure 1:**
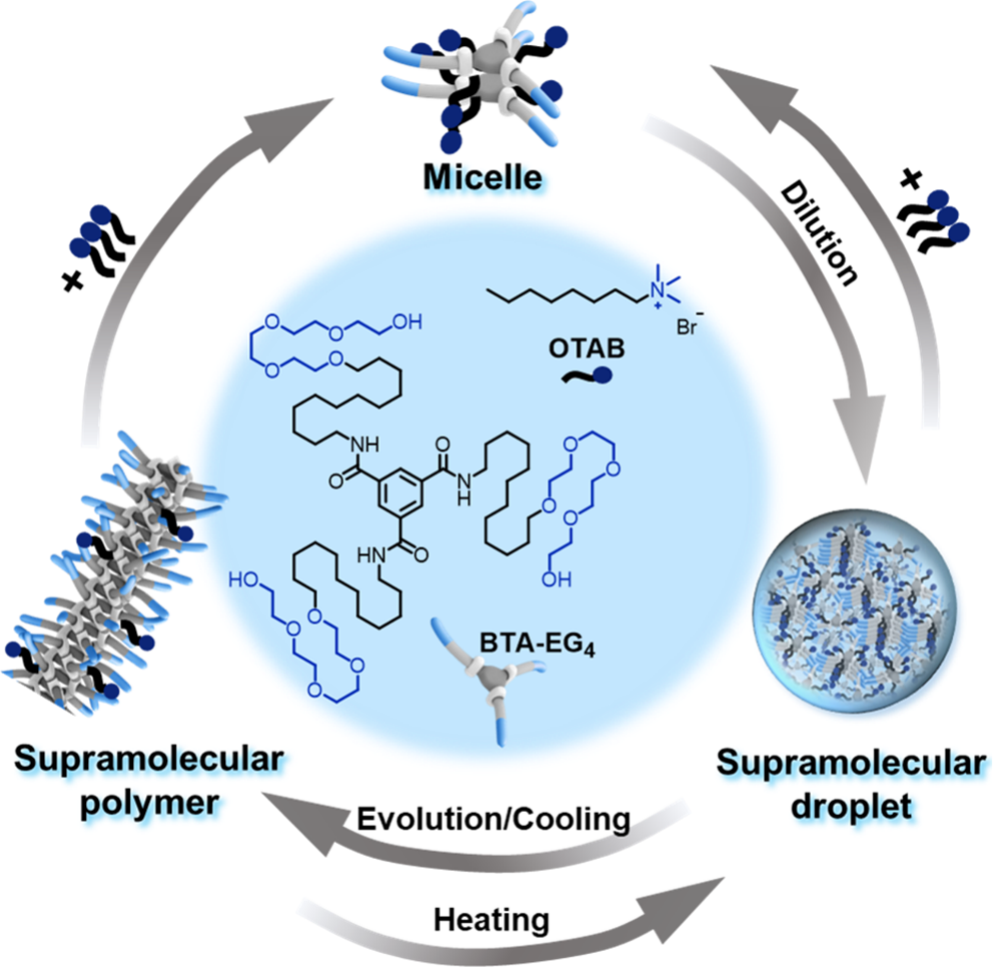
Schematic illustration of the supramolecular “micelle-droplet-fiber” transition of the BTA–OTAB system upon regulation of the competitive interactions.

We hypothesize that it is possible to regulate the supramolecular pathway between liquid droplets and the fibril state through competitive interactions due to their noncovalent nature. To explore this hypothesis, we propose a model system composed of highly dynamic small molecules that can be easily manipulated and studied by microscopy and spectroscopy. We selected the highly dynamic benzene-1,3,5-tricarboxamide motif, functionalized with amphiphilic tails composed of dodecyl and tetraethylene glycol (BTA-EG_4_, BTA in short hereafter, Figure 1), as it forms one-dimensional fibers driven by π–π stacking and hydrogen bonds, and it is capable of reorganizing in response to stimuli in water.^32−37^ We serendipitously observed that introducing the surfactant cetrimonium bromide (CTAB) into BTA supramolecular polymers can break down the long fibers and lead to complete disassembly at an optimized ratio and concentration.^38^ The developed thermodynamic mass-balance model described the importance of competition among various processes such as supramolecular polymerization, micellization, and complexation of the surfactant to the polymers.^38^ Interestingly, supramolecular polymerization is reinstated through an intermediate liquid droplet state upon dilution. We hypothesize that this reversibility originates from competitive interactions among the three components: BTA, the surfactant, and water. Therefore, in this study, we demonstrate the role of competitive interactions in regulating condensate dynamics and fibril formation with the BTA–surfactant model system with a concentration gradient field in water (Figure 1).

## Results and Discussion

### Rational Design

To explore the energy landscape of the BTA–surfactant system, we used octyltrimethylammonium bromide (OTAB) as the surfactant. Due to the shorter alkyl chain length of OTAB compared to CTAB, it exhibits weaker hydrophobic interactions with BTA. This allows us to slow down the dynamics of the system at millimolar (mM) concentration regimes, thereby expanding the operational window and fine-tuning the transitions. This tunability is critical for exploring the mechanistic aspects of the supramolecular phase transition and its regulation by competitive interactions. BTA and Cyanine 5-labeled BTA (BTA-Cy5, Figure S1) for fluorescence imaging were synthesized and characterized following previous reports.^32,39^

### Supramolecular “Micelle-Droplet-Fiber” Transition Using a Concentration Gradient Field

**Figure 2:**
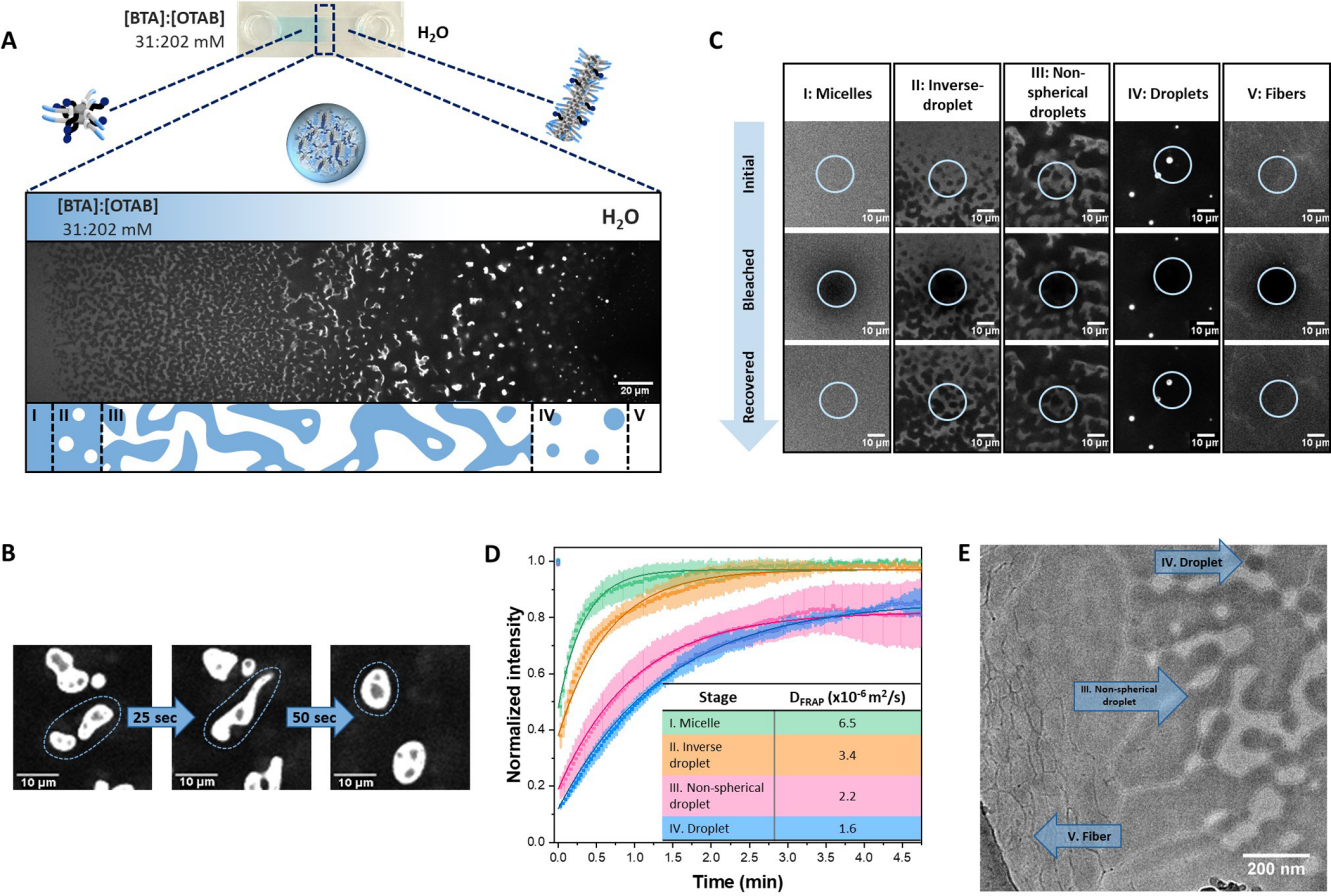
Supramolecular “micelle-droplet-fiber” transition using a concentration gradient field in the BTA–OTAB system. (A) CLSM image through a dual-inlet channel of a concentration gradient from [BTA]:[OTAB] (31:202 mM with 25 μM BTA-Cy5) to MQ water, which induces supramolecular “micelle-droplet-fiber” transition at the interface; scale bar = 20 μm. Below, a schematic overview is given to compare the shape of the droplets with the processes of nucleation and growth and spinodal decomposition. (B) CLSM images of droplet coalescence over time, indicated with a dashed circle; scale bar = 10 μm. (C) CLSM images of the various supramolecular structures before and after fluorescent bleaching at different stages (I–V) of the BTA–OTAB system; scale bar = 10 μm. (D) Exponential fitting of FRAP curves of stages (I–IV) with the corresponding *D*_FRAP_. (E) Cryo-TEM image of the BTA–OTAB turbid solution showing a phase separation pattern with a mixture of spherical droplets, nonspherical droplets, and 1D fibers; scale bar = 200 nm.

To create a concentration gradient of BTA–OTAB, we use a dual-inlet channel by introducing [BTA]:[OTAB] solution (31:202 mM with 25 μM BTA-Cy5, *sample preparation method II*) and milli-Q (MQ) water from the two inlets separately (Figure 2A). We investigated the effect of this concentration gradient with confocal laser scanning microscopy (CLSM). Initially, BTA–OTAB is a homogeneous micellar solution with the C_8_ aliphatic chain of OTAB inserted into BTA hydrophobic pockets.^38^ Dilution of the micellar phase weakens BTA–OTAB interactions, resulting in the partial release of OTAB from BTA–OTAB micelles and promoting LLPS. At the interface of the two solutions, supramolecular droplets were observed (Figures 2A and S2). Interestingly, these droplets appeared in both spherical and nonspherical shapes, which can be explained by the polymer phase diagram as predicted by Flory–Huggins theory.^40−43^ The nonspherical droplet shapes and inhomogeneity indicate that the interfacial tension between the droplets and the solvent is not yet minimized,^44^ which is due to the system being under continuous dilution. The fast change in the system leads to continuous new phase equilibria. The nonfluorescent region in the droplets corresponds to the BTA-sparse phase (Movies S1–S3). These droplets can coalesce (Figure 2B, Movie S1), confirming their liquid properties. Fluorescence recovery after photobleaching (FRAP) was conducted within the spherical droplets, and the rapid recovery of fluorescent signals further validates their liquidlike nature (Figure S2). Toward the water inlet, micrometer-long fibers were present (Figure S3) as a result of the stacking of newly exposed BTA. We identified five different stages across the interface within the BTA–OTAB system: micelles (I), inverse droplets (II), nonspherical droplets (III), droplets (IV), and fibers (V) (Figure 2A). Inverse droplets (II) refer to the droplets formed by BTA-sparse phase dispersed in the BTA-dense phase. The spherical droplets (II and IV) emerge from nucleation and growth, while the nonspherical droplets (III) either emerge from spinodal decomposition^45^ or result from the growth of inverse droplets (II).^46^ Cryogenic transmission electron microscopy (cryo-TEM, *sample preparation method VI*) showed a similar phase transition pattern with a mixture of spherical droplets, nonspherical droplets, and fibers at the interface layer (Figures 2E and S4) and densely packed fibers at the water layer (Figure S5).

FRAP was performed at different stages to evaluate the dynamics of the BTA–OTAB supramolecular structures (Figure 2C). All fluorescent signals recovered within approximately 2 min, which is faster than the recovery of BTA fibers alone (15% recovery in 4 min, Figure S6), indicating that OTAB significantly interferes with and weakens the BTA–BTA interactions. The apparent diffusion coefficients (*D*_FRAP_) of each state, derived from the fitted recovery curves, showed a consistent decrease from 6.5 × 10^–6^ m^2^/s for the micelles (I) to 3.4 × 10^–6^ m^2^/s for the inverse droplets (II), 2.2 × 10^–6^ m^2^/s for the nonspherical droplets (III), and eventually 1.6 × 10^–6^ m^2^/s for the droplets (IV) (Figure 2D), indicating a slowdown in dynamics. This trend can be attributed to an increase in intermonomer and/or interoligomer interactions, driven by the gradual release of OTAB and the greater exposure of BTA hydrophobic pockets. Additionally, the decrease in the concentration from the inlets slows diffusion. Near the inlet, where the concentration of micelles is the highest, recovery occurs more rapidly. However, as the droplet concentration decreases with the distance from the inlets, the recovery rate slows, primarily due to a decrease in diffusion. Fibers (V) are expected to have an even slower diffusion, yet a relatively fast recovery with a *D*_FRAP_ of 2.8 × 10^–6^ m^2^/s was observed, probably due to the presence of OTAB in the BTA fiber networks (Figure S6).

As control experiments, replacing MQ water with a 16 or 31 mM OTAB solution resulted in transient phase separation, while higher OTAB concentrations (47, 101, or 202 mM) showed neither phase separation nor fibers, but only a homogeneous micellar solution (Figures S7 and S8). This suggests that competitive interactions among the three components—BTA, surfactant, and water—modulate the order of the system through “micelle-droplet-fiber” transition. Therefore, in the following study, we investigate the molecular interaction in more detail.

### Supramolecular “Micelle-Droplet-Fiber” Transition over Time upon Dilution

**Figure 3:**
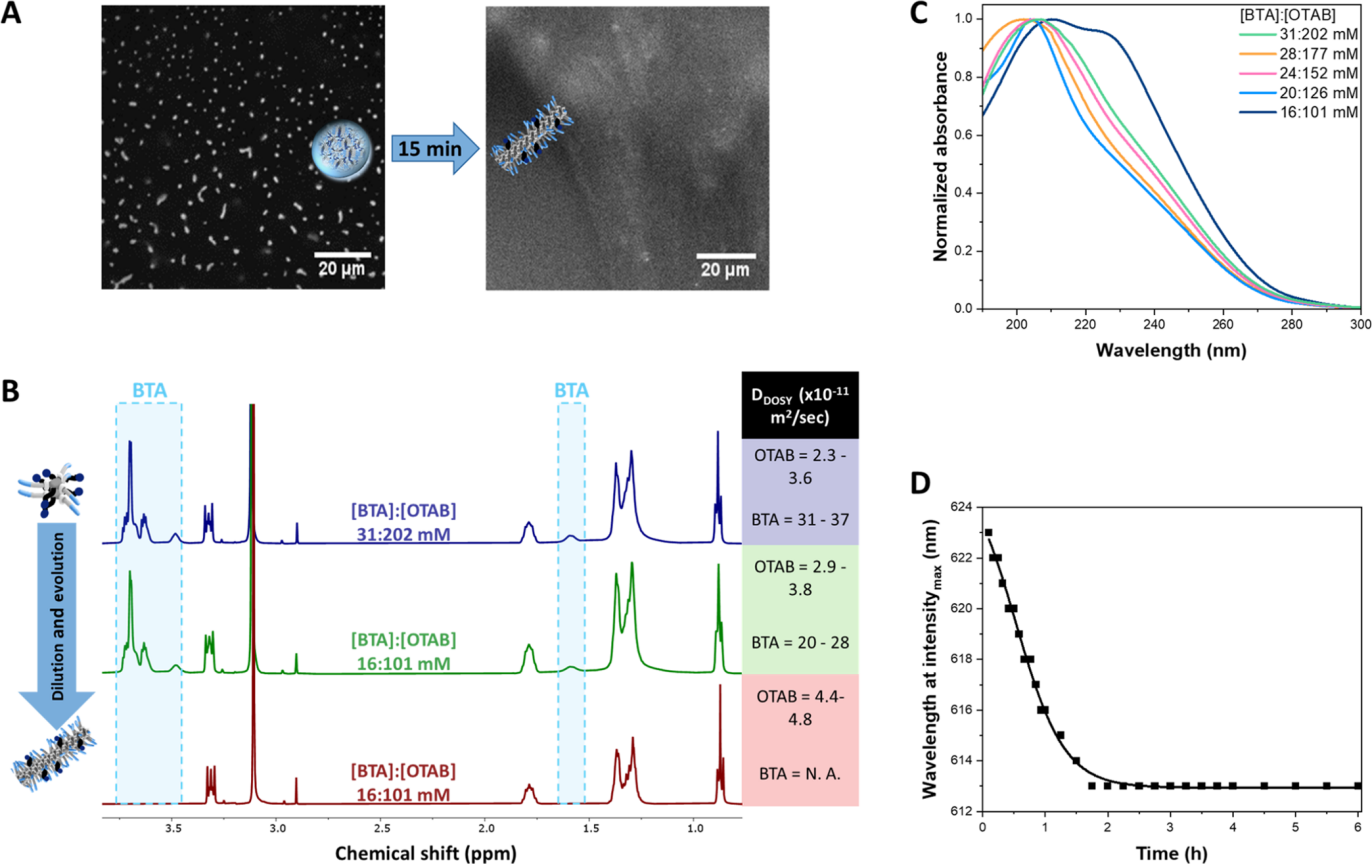
Temporal control of the dilution-induced supramolecular transition. (A) CLSM images of the transition from supramolecular droplets into a fiber network over 15 min at 22 °C; scale bar = 20 μm. (B) Stacked ^1^H NMR spectra of [BTA]:[OTAB] micelles (31:202 mM), supramolecular droplets (16:101 mM), and fibers (16:101 mM) in D_2_O with their corresponding *D*_DOSY_. (C) UV spectra of different concentrations of [BTA]:[OTAB], yet with a fixed ratio (*sample preparation method III*). (D) Time-dependent fluorescence emission of Nile red in freshly prepared [BTA]:[OTAB] (16:101 mM, *sample preparation method II*).

The time evolution of BTA–OTAB morphologies upon dilution was initially observed by CLSM, where the droplets faded and converted into a fiber network within approximately 15 min at 22 °C (Figure 3A), whereas the droplets maintained their shape for >1 h at 37 °C (Movie S2). At lower temperatures, fiber formation is more thermodynamically favored, while at higher temperatures, the monomers are more dynamic, hindering supramolecular polymerization, which results in the prolonged stability of the droplets.^47^ This allows us to manipulate the landscapes of the transition with temperature in the latter study. Nevertheless, capturing the droplets exclusively is challenging as they start to convert into fibers upon mixing. We attempt to understand these intermediate states, which are probably a mixture of droplets and short fibers. To gain a deeper mechanistic insight into this “micelle-droplet-fiber” transition, we employed a combination of proton and diffusion-ordered nuclear magnetic resonance spectroscopies (^1^H and DOSY NMR) and ultraviolet–visible (UV–vis) and Nile red fluorescence spectroscopies, together with cryo-TEM (Figures 3 and S9–S20). For all NMR measurements, an external standard, 3-(trimethylsilyl)propionic-2,2,3,3-*d*_4_ acid sodium salt (TPS), in a capillary NMR tube, was employed to ensure proper alignment (chemical shift = 0.0 ppm, Figures S9–S12) and to serve as an indicator for the amount of portable OTAB.

As depicted in Figure 3B, the ^1^H NMR spectrum of the initial micellar state ([BTA]:[OTAB] = 31:202 mM, s*ample preparation method I*) showed sharp and well-split peaks for both BTA (signals in blue boxes) and OTAB. Upon a 2-fold dilution, the micellar solution immediately became visually turbid, indicating phase separation and the formation of droplets corresponding to stages II–IV (Figure S13). The NMR peaks corresponding to OTAB remained sharp and well resolved. The peaks associated with BTA showed slight broadening and a decrease in integrals, with the most significant reduction from the aliphatic chain (Table S1), suggesting that the hydrophobic interactions of the C_12_-linker initially drive phase separation. After vigorous mixing and a 24 h incubation, the BTA peaks disappeared due to the slow diffusion of the large BTA fibers, as corroborated by cryo-TEM (Figure S14). The OTAB integral decreased by approximately 10%, indicating that these 10% OTAB molecules were associated with the BTA fibers.

We subsequently assessed the translational diffusion coefficients (*D*_DOSY_) of the assemblies using DOSY NMR (Figures 3B and S15–S18). Due to the fast exchange and high dynamics, two interesting phenomena were observed: (i) The *D*_DOSY_ of BTA and OTAB were always in two distinct populations throughout the dilution and evolution processes. (ii) The *D*_DOSY_ of both BTA and OTAB were no single values but fell within narrow regimes. Specifically, the *D*_DOSY_ of OTAB increased as it transitioned from the micellar state to the supramolecular droplets and eventually to the fiber state. This faster diffusion is attributed to OTAB being gradually expelled from the BTA hydrophobic pockets, which weakens the BTA–OTAB interaction. In contrast, the *D*_DOSY_ of BTA decreased upon dilution, reflecting the slower diffusion of the forming droplets/short fibers, driven by the enhanced BTA–BTA interaction. In the final micrometer-long fiber state, the BTA signal became nondetectable.

The UV spectra at decreasing concentrations of [BTA]:[OTAB] with a constant ratio showed a red shift in the absorption, from a characteristic micellar state ([BTA]:[OTAB] = 31:202 mM) with a maximum absorption (λ_max_) around 204 nm to a BTA double-helix fiber state ([BTA]:[OTAB] = 16:101 mM) with maxima of 211 and 227 nm (*sample preparation method III*; Figures 3C and S19).^48^ Nile red fluorescence spectroscopy further demonstrated a blue shift of emission λ_max_ from 623 to 613 nm, indicating that Nile red is experiencing a more hydrophobic environment over time (*sample preparation method II;*Figures 3D and S20).^18^

### Reversibility of Fiber-to-Droplet and Droplet-to-Micelle Transitions Using Competitive Interactions

With an understanding of the competitive interactions to regulate the supramolecular “micelle-droplet-fiber” transition, we subsequently investigate different strategies based on these competitive interactions as a general concept to modulate the supramolecular landscapes of these transitions.

### Reversible Fiber-to-Droplet Transition

**Figure 4:**
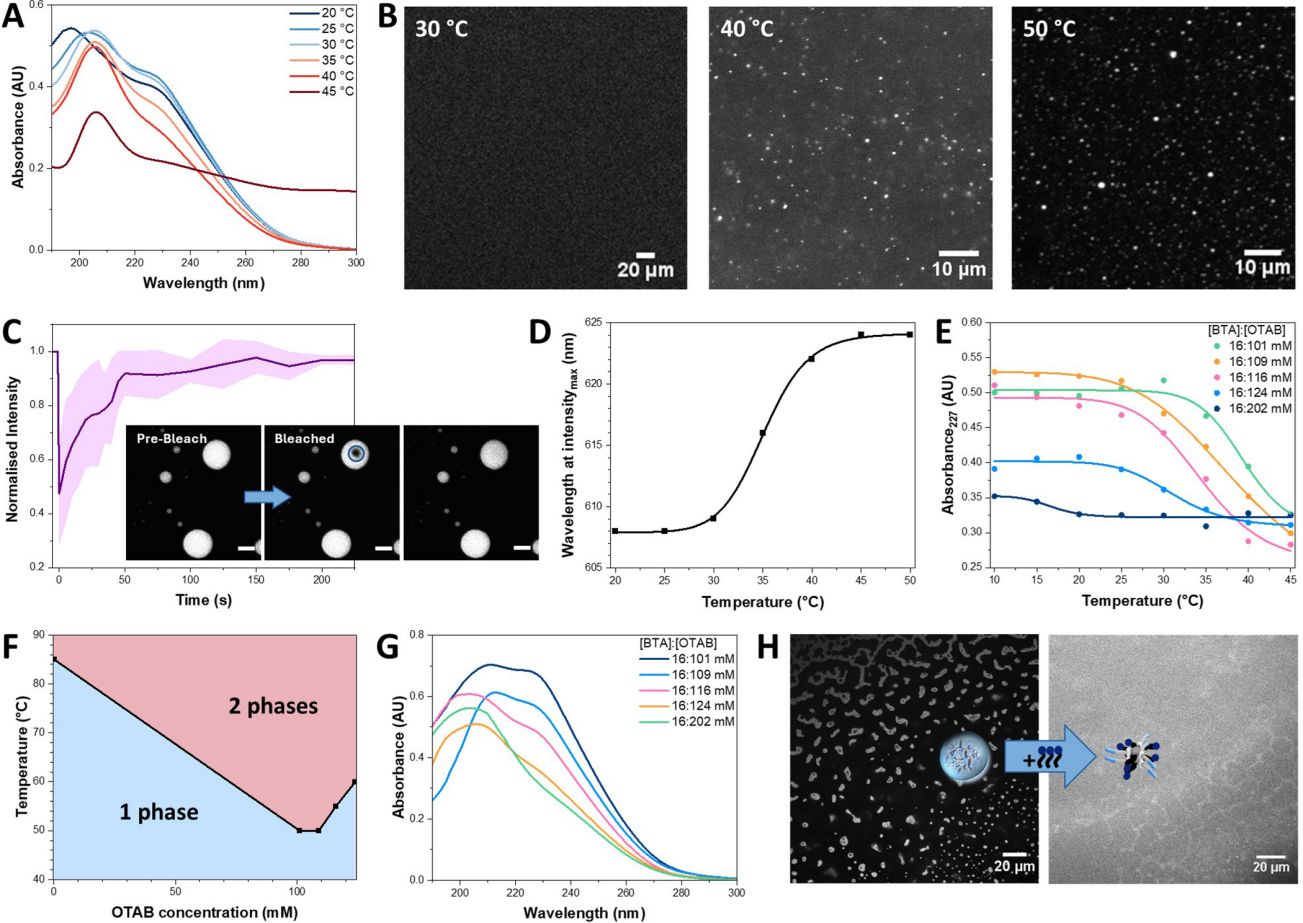
Reversibility of fibers and droplets by tuning competitive interactions. (A) Temperature-dependent UV spectrum of [BTA]:[OTAB] 16:101 mM (*sample preparation method V)*. (B) Temperature-dependent CLSM images of [BTA]:[OTAB] 16:101 mM with 25 μM BTA-Cy5 (*sample preparation method V)*; scale bar = 20 or 10 μm. (C) CLSM images during FRAP at 40 °C and the corresponding normalized FRAP kinetics; scale bar = 10 μm. (D) Temperature-dependent fluorescence emission of [BTA]:[OTAB] 16:101 mM with 20 μM Nile red (sample preparation method V). (E) Absorbance at 227 nm of several [BTA]:[OTAB] ratios by varying the temperature from 10 to 45 °C (*sample preparation method IV)*. (F) Phase separation temperature at several [BTA]:[OTAB] ratios during the heating process (*sample preparation method IV)*. (G) UV spectra of [BTA]:[OTAB] with varying OTAB concentrations (*sample preparation method IV)*. (H) CLSM images of [BTA]:[OTAB] 31:202 mM droplets in a concentration gradient by a dual-inlet chamber system, before and after addition of 202 mM OTAB; scale bar = 20 μm.

We recently observed that the BTA fiber itself started depolymerizing at 60 °C and initiated droplet formation above 80 °C, owing to the dehydration of tetraethylene glycol.^18^ Remarkably, introducing [OTAB] = 101 mM in the BTA system ([BTA] = 16 mM) at room temperature significantly decreases the onset temperature of depolymerization to 35 °C and fiber-to-droplet transition to 45 °C, as indicated by the scattering effect at 300 nm (*sample preparation method V*; Figures 4A and S21). This was also confirmed with CLSM that the fiber network started to convert to droplets at 40 °C, and a complete transition to droplets was displayed at 50 °C (*sample preparation method V*; Figure 4B). The FRAP experiment confirmed the liquid nature of these droplets formed at 40 and 50 °C (Figures 4C and S22). The decreased phase separation temperature from 80 to 40 °C can be attributed to the weakened BTA–BTA interactions where OTAB disturbs the one-dimensional ordered arrangement of the BTA core. Thereof, the fibers are broken down into short fibers; meanwhile, exposure of the hydrophobic pockets serves as a driving force for the formation of droplets. To further confirm the fiber-to-droplet transition, temperature-dependent fluorescence emission spectra were recorded with Nile red that was embedded in a mixture of the BTA–OTAB fiber state (*sample preparation method V*; Figures 4D and S23). Depolymerization and droplet formation were verified by a red shift of the emission λ_max_ from 608 to 623 nm, while the λ_max_ of Nile red in water as a control showed a minor blue shift of 2 nm (Figure S23E).

The BTA:OTAB ratio effect in the fiber-to-droplet transition was further investigated by variable-temperature UV–vis spectroscopy. While [BTA] was fixed at 16 mM, [OTAB] was gradually increased beyond 101 mM and samples were annealed (*sample preparation method IV)*. The onset temperature of depolymerization decreased from approximately 34 to 14 °C (Figures 4E and S24), while the phase transition temperature increased from 50 to 60 °C for [OTAB] from 101 to 124 mM (s*ample preparation method IV*; Figures 4F and S24). We hypothesize that above a critical OTAB ratio, the hydrophobic BTA–BTA interactions are completely overcome by addition of the cationic charge of OTAB. This repulsive interaction decreases the depolymerization energy barrier. In addition, the droplet state is majorly driven by the dehydration of tetraethylene glycol, resulting in a higher onset temperature of phase separation.

### Fiber-to-Micelle Transition

On increasing the concentration of OTAB, a fiber-to-micelle transition occurs (s*ample preparation method IV*; Figures 4G and S25).^38^ At [BTA]:[OTAB] = 16:202 mM, a complete micellar state was reached, and no depolymerization or phase separation was detected even up to 80 °C (Figure S26E). This is depicted by the UV spectra showing a characteristic micellar absorption at 204 nm.

### Droplet-to-Micelle Transition

Interestingly, the micelle-to-droplet transition is observed by introducing additional OTAB. This is observed in CLSM images, where supramolecular droplets at the interface of [BTA]:[OTAB] (31:202 mM) to H_2_O in the dual-inlet chamber system converted to a homogeneous micellar solution upon adding OTAB solution in the water phase (*sample preparation method II*; Figure 4H).

To generalize the role of competitive interactions in determining the supramolecular state, we evaluated the solvent polarity using a methanol–water mixture. [BTA]:[OTAB] (31:202 mM) and 5% or 8% methanol in water were injected in either inlet of the channel (Figure S27). It was observed that the supramolecular droplet or fiber formation can be inhibited by the competitive interaction of 8% methanol. At 5% methanol, still some traces of supramolecular droplets were observed at the interface of the two solutions.

## Conclusions

In this study, we demonstrate the role of competitive interactions in determining the supramolecular landscape and the extent of order in the system. Due to the dynamic and noncovalent nature of BTA assemblies, the concentration of the competitive guest and temperature regulate the micelle, droplet, or polymer state, as well as their transition points. Through this model system, we reiterate that balances among various interactions are crucial. At the molecular level, these are interactions between the monomer, the guest, and the solvent, not only at the monomer core but also at the tails. At the supramolecular level, these are the intermonomer and interfiber interactions that influence the pathway of assembly and disassembly. However, expanding this understanding from simple synthetic molecules with limited possibilities of intermolecular interactions to complex macromolecules, such as proteins, requires comprehensive studies using combined experimental and theoretical approaches. This would provide us with a large database that can use artificial intelligence to predict the required competing guests or key interprotein interactions that need to be targeted. These key interprotein interactions need special attention for mechanistic understanding and drug designing for condensate-modifying therapeutics.^49−51^

## Supporting Information Available

CLSM video of BTA-OTAB merging droplets (AVI)
CLSM video of BTA-OTAB droplets maintain the shape (AVI)
CLSM video of BTA-OTAB elongated into spherical droplet (AVI)
Experimental details, supporting figures of fluorescent microscopy, NMR characterization, spectroscopic characterization, and cryo-TEM images (PDF)

## Supplementary Material

ja4c11285_si_001.avi

ja4c11285_si_002.avi

ja4c11285_si_003.avi
