# Supplementary material for: Harnessing Competitive Interactions to Regulate Supramolecular “Micelle-Droplet-Fiber” Transition and Reversibility in Water
Source: J Am Chem Soc. 2024 Oct 15;146(43):29759–66. doi: 10.1021/jacs.4c11285 (PMC11528417; doi:10.1021/jacs.4c11285)
Supplement: Supplementary file 4 — ja4c11285_si_004.pdf [file ja4c11285_si_004.pdf]

Supplementary information

Harnessing competitive interactions to regulate supramolecular "micelle-droplet-fiber" transition and reversibility in water

Heleen Duijs,[a] Mohit Kumar,[b] Shikha Dhiman,*[b] and Lu Su*[a]

[a]Division of Biotherapeutics, Leiden Academic Centre for Drug Research (LACDR), Leiden University, Einsteinweg 55, 2333 CC Leiden, The Netherlands.

[b] Department of Chemistry, Johannes Gutenberg University in Mainz, Duesbergweg 10-14, D-55128 Mainz, Germany.

Table of content

1. Experimental procedures **3**

1.1 Materials *3*

1.2 Sample preparation *3*

1.3 Confocal laser scanning microscopy (CLSM) *3*

1.4 Total internal reflection fluorescence (TIRF) microscopy *4*

1.5 Fluorescent recovery after photobleaching (FRAP) *4*

1.6 Cryogenic transmission electron microscopy (cryo-TEM) *4*

1.7 Nuclear magnetic resonance (NMR) spectroscopy *4*

1.8 Ultraviolet visible (UV-vis) spectroscopy *4*

1.9 Fluorescence spectroscopy *5*

2. Supplementary figures **6**

3. References **22**

1. Experimental procedures

1.1 Materials

Trimethyloctylammonium bromide (OTAB) was purchased from Sigma-Aldrich and 3-(trimethylsilyl)propionic-2,2,3,3-d_4 acid sodium salt (TSP) from Euriso-top (Park of algorithms, St-Aubin Cedex), both used without further purification. BTA and BTA-Cy5 were synthesized, according to literature procedures.[1,2] The samples were dissolved in Milli-Q H_2O (Milli-Q EQ 7000, Merck Millipore, MQ H_2O). The dual-inlet channel (μ-Slide VI 0.5 Glass Bottom) for confocal laser scanning microscopy (CLSM) and total internal reflection fluorescence (TIRF) microscopy were obtained from Ibidi.

1.2 Sample preparation

Since sample preparation can have a large effect on the dynamics and morphology, sample preparation methods are clarified below. All CLSM samples were prepared according to according to s*ample preparation method II,* unless stated otherwise. All NMR samples were prepared according to s*ample preparation method I.*

Sample preparation method I: Appropriate amounts of BTA and OTAB were dissolved in MQ H_2O. The mixture was stirred for 15 min at 90 °C, followed by equilibration overnight at room temperature. Afterwards the samples were stored at 4 °C.

Sample preparation method II: For fluorescent imaging, the BTA-OTAB solution was prepared as stated in *sample preparation method* with the addition of 25 μM BTA-Cy5 after heating at least 24 h before imaging. For fluorescence spectroscopy, 20 μM Nile red (from 3.14 mM stock solution in methanol) was added to the BTA-OTAB sample after heating and incubated for 1 h.

Sample preparation method III: UV-vis samples at different dilutions of [BTA-OTAB] for UV-vis were prepared by diluting [BTA]:[OTAB] (31:202 mM), prepared as mentioned in *sample preparation method I*, to the desired concentration and measured after 24 h equilibration at room temperature.

Sample preparation method IV: UV-vis samples with varying ratios of [BTA]:[OTAB] (with [BTA] = 16 mM) were prepared by dissolving BTA and OTAB in the desired amount in MQ. This mixture was heated to 90 °C for 15 min, followed by at least overnight equilibration.

Sample preparation method V: For kinetically trapped UV-vis and fluorescence spectroscopy, 31 mM BTA was prepared by heating to 90 °C for 15 min, followed by equilibration overnight at room temperature. Then, an equal volume of 202 mM OTAB was added and 20 μM Nile red (from 3.14 mM stock solution in methanol) in case of fluorescent measurements. This mixture was vortexed for 1 min and measured immediately. For CLSM 25 μM BTA-Cy5 was added after heating.

Sample preparation method VI: Cryogenic transmission electron microscopy (cryo-TEM) samples were prepared in a PCR Eppendorf tube (0.2 mL), where 10 μL of [BTA]:[OTAB] (31:202 mM) micellar solution was gently added into 10 μL of MQ H_2O. The turbid interface layer (2 μL) or the water layer (2 μL) was immediately applied to fresh glow-discharged Quantifoil® grids (R 2/2, Quantifoil Micro Tools GmbH) or Lacey grids (LC200-Cu-150, Electron Microscopy Sciences).

1.3 Confocal laser scanning microscopy (CLSM)

Confocal microscopy was conducted on a Leica TCS SP8 and Nikon Eclipse Ti inverted confocal microscope with temperature regulation by OKO lab incubator. An 60x oil-immersion objective (Nikon Plan Apo 60×/ 1.40 Oil DIC N2) and was utilized. The samples were excited with laser λ_{ex} = 640 nm for Cy5 excitation and emission was detected within the window of λ_{em} = 663-738 nm. Ibidi μ-Slides VI 0.5 Glass Bottom were used to create the concentration gradient. In these channels first 16 μL of aqueous solution was injected, followed by 16 μL of the BTA-OTAB solution with BTA-Cy5. Images were generated close to the bottom surface of the slide. Confocal images were processed in Fiji ImageJ.

1.4 Total internal reflection fluorescence (TIRF) microscopy

TIRF images were acquired with a Nikon N-STORM microscopy system. Sample was excited using 561 nm laser. Fluorescence was collected using a NikonÅ~100, 1.4NA oil immersion objective and passed through a quad-band pass dichroic filter (97335 Nikon). Images were recorded with an EMCCD camera (ixon3, Andor, pixel size 0.17 μm).

1.5 Fluorescence recovery after photobleaching (FRAP)

Using the set-up mentioned in section *1.3 Confocal laser scanning microscopy (CLSM)*, FRAP was measure within a region of interest with a diameter of 14 μm. The region of interest was subjected to 100% beaching power of the λ_{ex}=633 nm laser for 20 seconds. Then, every 2 seconds an image was recorded for 5 minutes. Three FRAP experiments were conducted for each condition and averaged. Image analysis for drift correction and quantification of fluorescent intensity was performed in Fiji ImageJ, according to literature procedures.[3,4] The fluorescent intensity was normalized by intensity of a reference area, following equation (i), where *i*(t) is the normalized fluorescent intensity at time *t*, *I(t)* and *I(0)* are the fluorescence intensities of the region of interest at time *t* and *t*=0.

$$i\,(t) = \frac{I(t)/R(t)}{I(0)/R(0)} \qquad \text{(i)}$$

In order to obtain the apparent diffusion coefficient (D_{app}), the recovery curves were fitted with first-order exponential equation (ii), where *i*(t) is normalized fluorescent intensity at time *t*, *A* is the amplitude of recovery, *τ* is the critical recovery time and *C* is the intercept.

$$i(t) = A\left(1 - e^{-\frac{t}{\tau}}\right) + C \qquad \text{(ii)}$$

D_{app} was calculated with equations (iii) and (iv), where $t_{1/2}$ is the half-life of recovery, *τ* is the critical recovery time and *ω* is radius of the region of interest.

$$t_{1/2} = ln2 \times \tau \qquad \text{(iii)}$$

$$D_{app} = \frac{0.88\omega^2}{4t_{1/2}} \qquad \text{(iv)}$$

1.6 Cryogenic transmission electron microscopy (cryo-TEM)

For cryo-TEM sample preparation, 2 μL of the freshly prepared BTA-OTAB turbid solution at the interface layer or transparent water layer (see *Sample preparation method VI*) was applied to fresh glow-discharged Quantifoil® grids (R 2/2, Quantifoil Micro Tools GmbH) or Lacey grids (LC200-Cu-150, Electron Microscopy Sciences). Grids were plunge-frozen in liquid ethane using a Vitrobot Mark IV (Thermo Fisher Scientific) set at 22 °C, 100% humidity, and 12 s blotting time, due to sample's high viscosity. Samples were imaged in a Talos L120C TEM (Thermo Fisher) equipped with a LaB6 filament operating at 120 kV accelerating voltage. Images were recorded using a Ceta camera.

1.7 Nuclear magnetic resonance (NMR) spectroscopy

^{1}H and DOSY NMR were recorded on a Bruker 500 MHz spectrometer in D_2O as solvent. As an external standard, 100 mM TPS was dissolved in D_2O in a capillary NMR tube and inserted in the NMR tube with BTA-OTAB to avoid interaction of TPS and BTA-OTAB. All measurements were performed at 25 °C and chemical shifts were expressed in parts per million (ppm). For DOSY, the diffusion time interval (d20 or D) was set at 200 ms and the gradient pulse strength (p30 or δ) at 1200 ms. The proton 90° pulse was 14.2 μs with -12.07 dB. The DOSYs measurements were recorded over 8 scans with a pulse gradient field strength varying from 2% to 95% in 16 steps. DOSY plots were acquired with MestReNova v14.3.3-33362 with DOSY/ROSY transform peak fit method.

1.8 Ultraviolet visible (UV-vis) spectroscopy

UV-vis measurements were conducted on an Agilent 8453 spectrometer with Agilent 89090A controller which is equipped with Peltier temperature control. The spectral range was chosen between 190 nm and

350 nm with 1 nm interval. The samples were prepared in MQ H_2O, according to protocol described in *1.2 sample preparation* section. These solutions were measured in a high precision cell of Quartz glass (Hellma Analytics) with pathlength of 0.01 mm together with the cell holder (for cell type 106, Hellma Analytics). OTAB at identical condition is used as blank for all measurements. After temperature adjustments, the sample was allowed to equilibrate until the absorbance remained stable, unless equilibration time is stated otherwise. For each condition three measurements were recorded and averaged. Data analysis was performed in OriginPro 2022 9.9.0.225 (Academic) for visualization and smoothing (Savitzky-Golay method) of the graphs.

1.9 Fluorescence spectroscopy

Emission spectra of 20 μM Nile red were recorded on a Fluorescence Spectrometer (FS 920, Edinburgh Instruments) with Xe900 lamp as light source and sample holder with temperature regulation. Samples were prepared as stated in the *1.2 sample preparation* section and measured in an ultra-micro cell for fluorescence of Quartz glass (Hellma Analytics) with pathlength of 3.0 mm. The sample was excited at λ_{ex} = 579 nm and the emission spectrum were recorded from λ_{em} = 590-750 nm. Time-dependent measurements were performed at 20 °C.

2. Supplementary figures

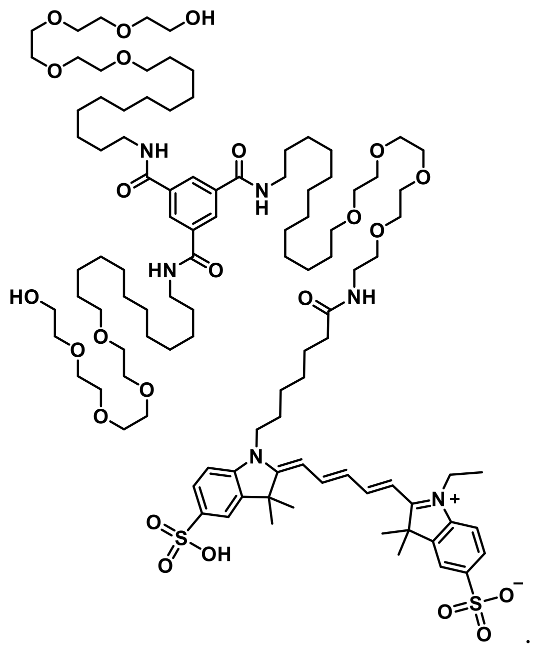

Figure S1: Chemical structure of BTA-Cy5.

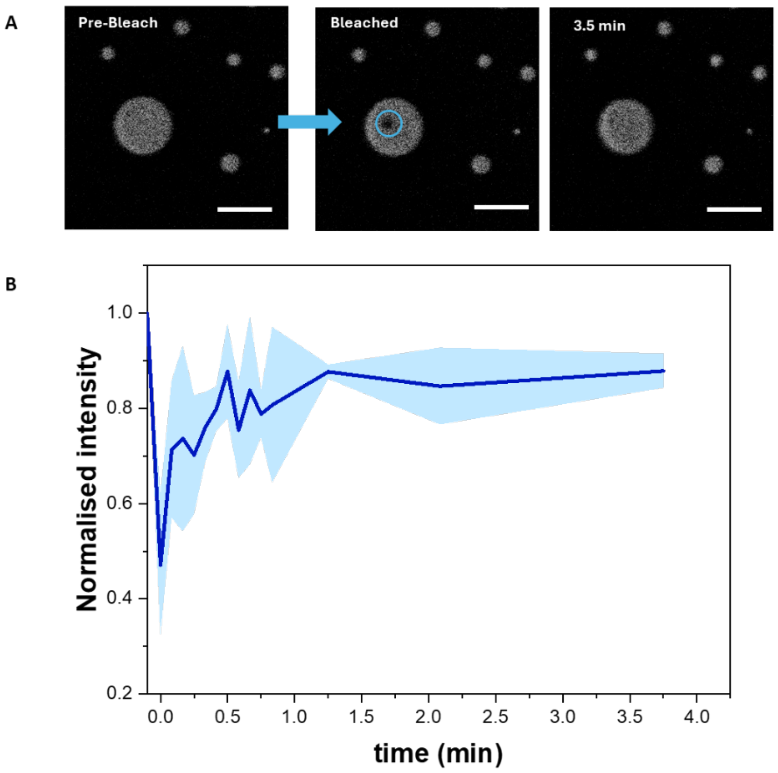

Figure S2: (A) CLSM images during FRAP. (B) Corresponding normalized FRAP kinetics. Scale bar = 10 μm

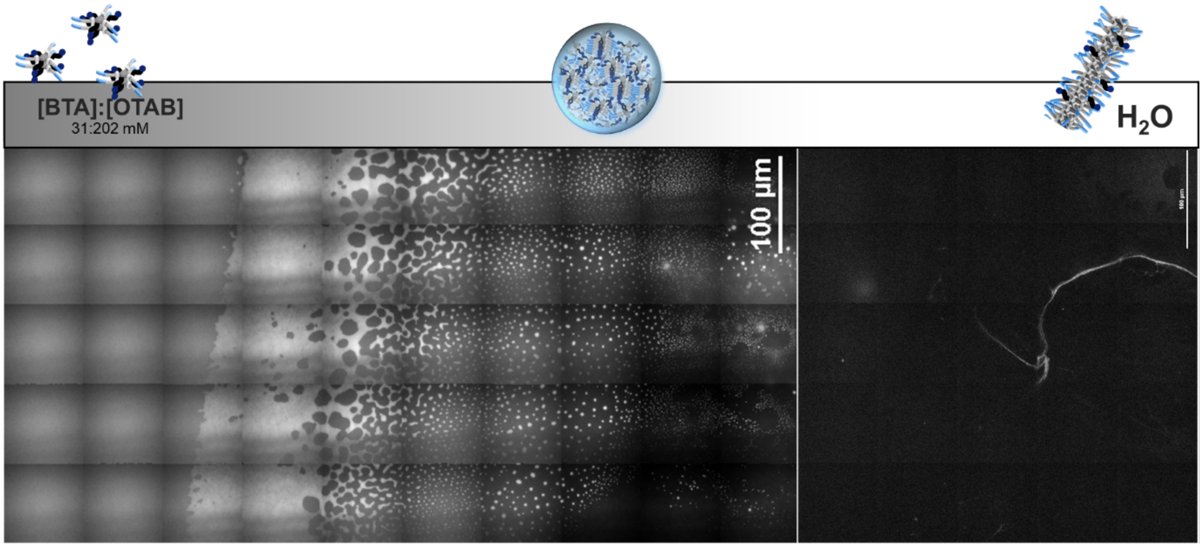

Figure S3: Micelles, supramolecular droplets and fibers visualized with TIRF confocal microscopy at 22 °C. A concentration gradient from [BTA]:[OTAB] (31:202 mM with Nile red) to MQ shows multiple supramolecular assemblies, evolving from micelles to supramolecular droplets to fibers.

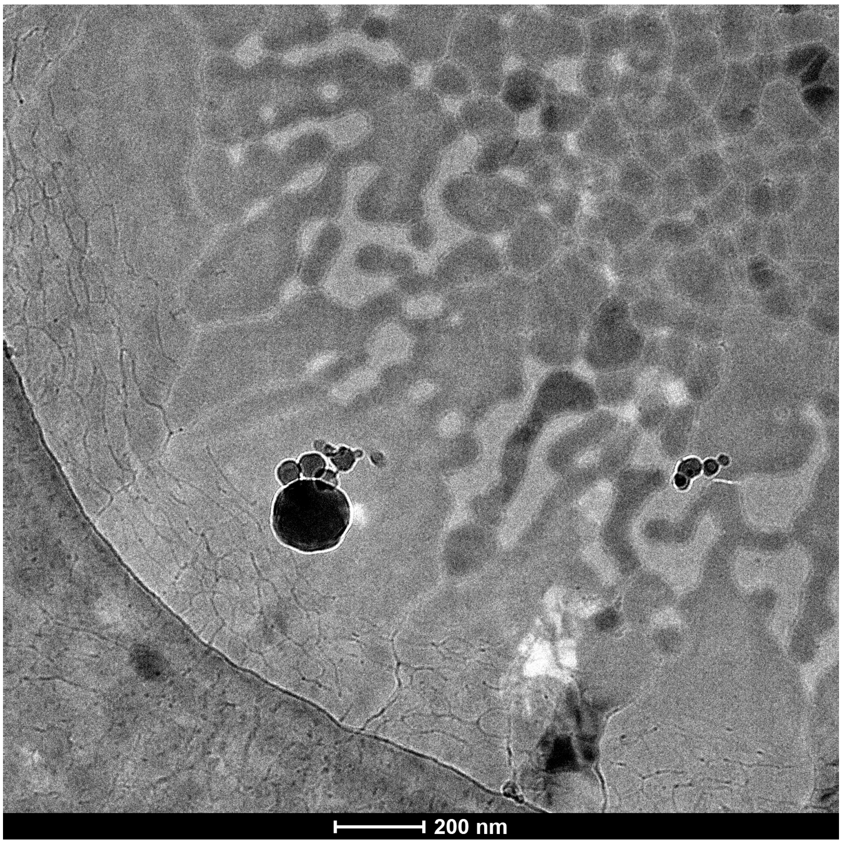

Figure S4: Cryo-TEM image of BTA-OTAB turbid solution (see *Sample preparation method VI*), showing the phase separation pattern with a mixture of spherical droplets, non-spherical droplets, and 1D fibers. The black spherical particles are ice-crystals. Scale bar = 200 nm.

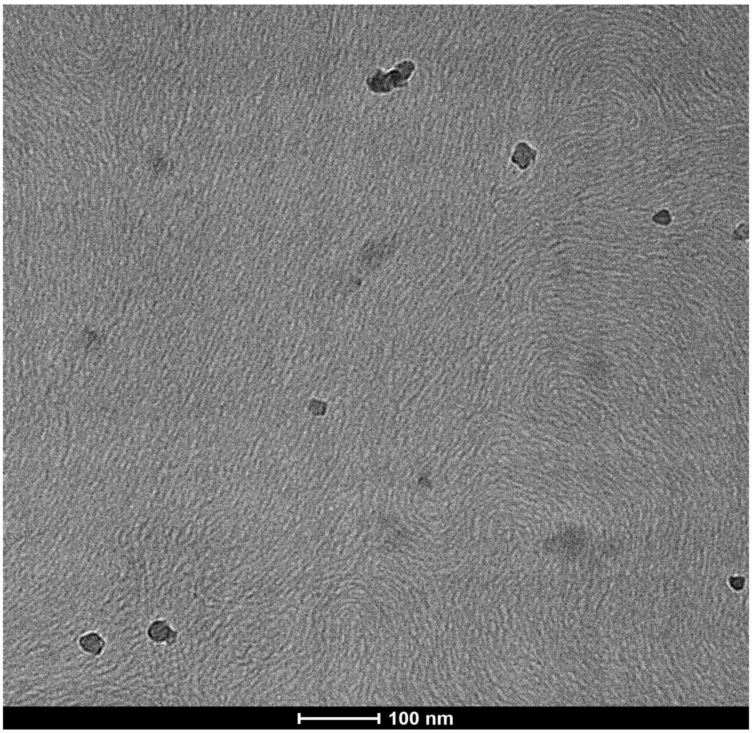

Figure S5: Cryo-TEM images of BTA-OTAB water layer (see *Sample preparation method VI*), showing densely packed micrometer long 1D fibers. The black spherical particles are ice-crystals. Scale bar = 100 nm.

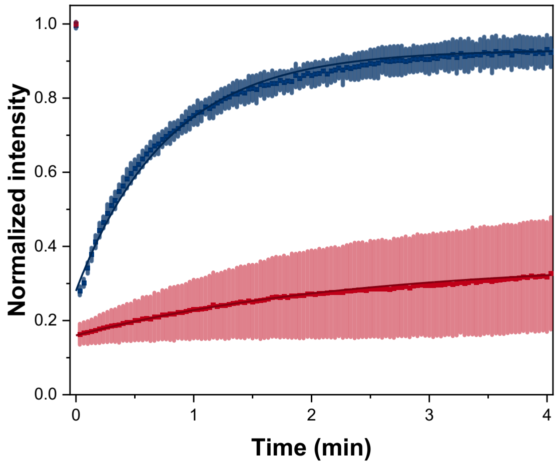

Stage	D_{FRAP} (x10^{-6} m^2/sec)
V. Fiber in concentration gradient	2.8
31 mM BTA	93

Figure S6: FRAP recovery curve of BTA-OTAB fiber networks in concentration gradient (blue) and 31 mM BTA (red) with corresponding diffusion coefficients.

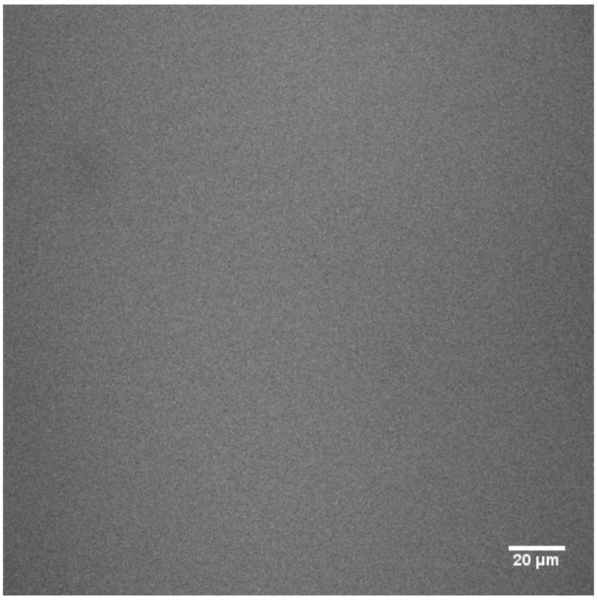

Figure S7: CLSM image at interface of gradient of [BTA]:[OTAB] (31:202 mM) to OTAB (202 mM) at 22 °C.

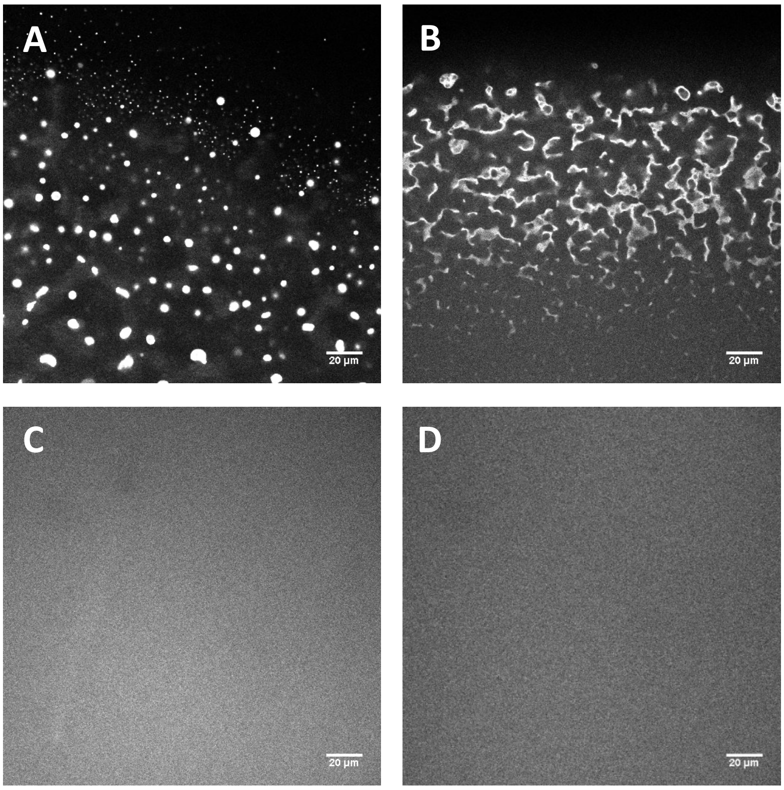

Figure S8: CLSM images of gradient of [BTA]:[OTAB] (31:202 mM) to OTAB (A) 16 mM, (B) 31 mM, (C) 47 mM and (D) 101 mM at 37 °C .

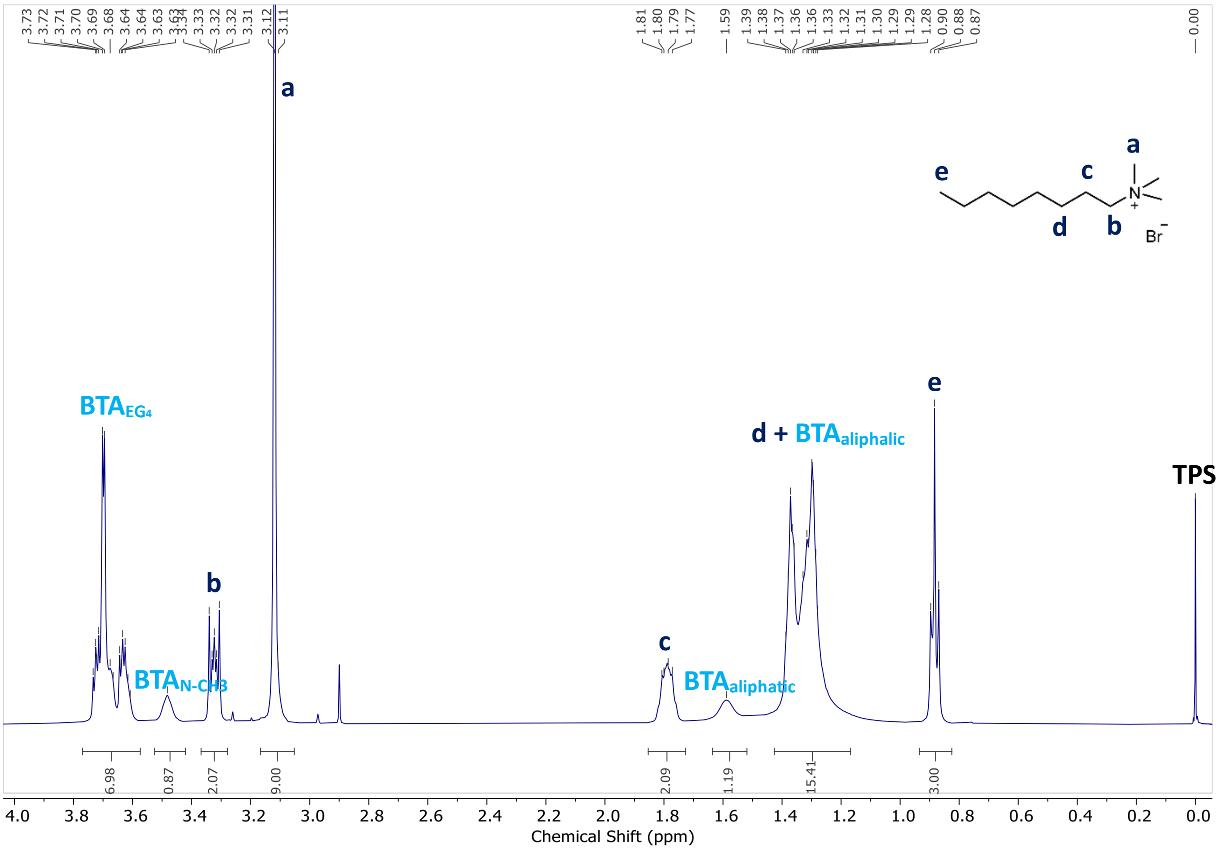

Figure S9: ^{1}H NMR spectrum of the micellar solution of [BTA]:[OTAB] (31:202 mM) in D_2O recorded at 500 MHz, 25 °C.

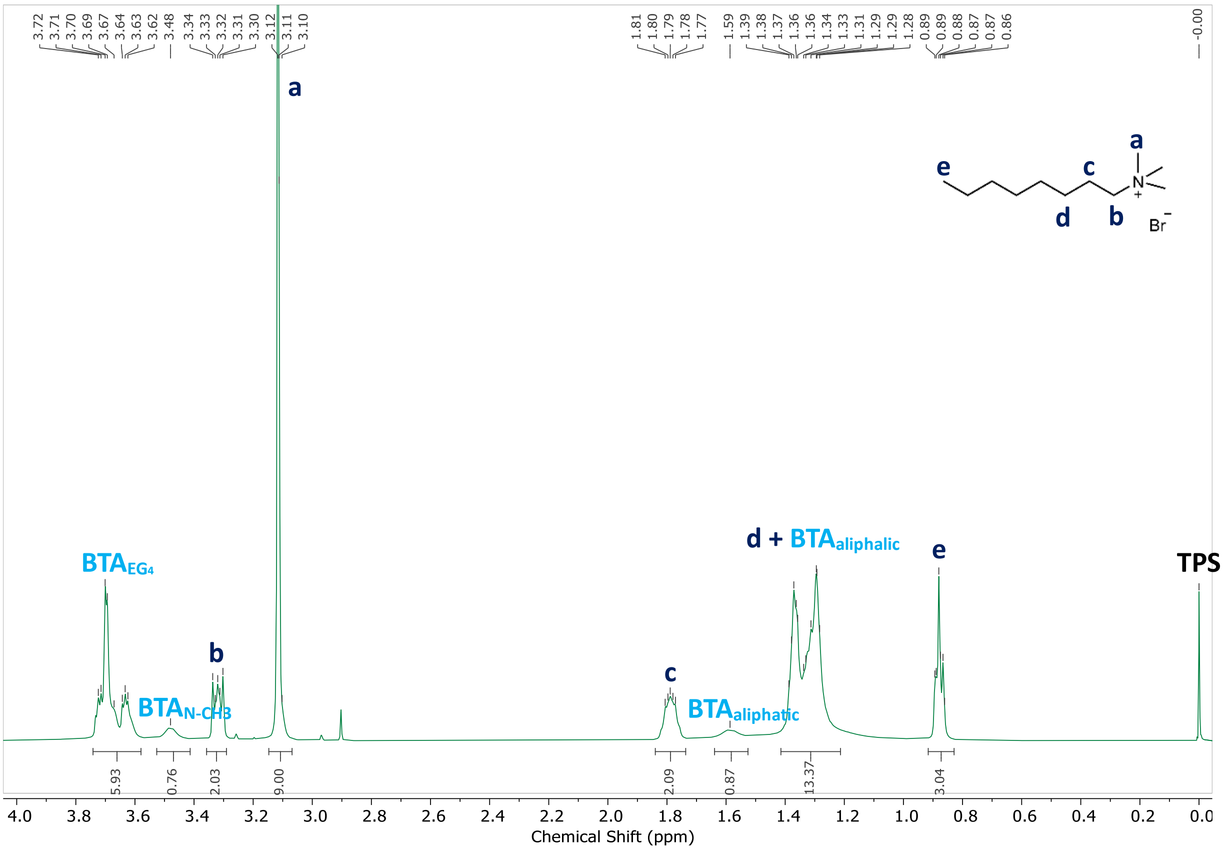

Figure S10: ^{1}H NMR spectrum of [BTA]:[OTAB] (16:101 mM) directly after dilution in D_2O recorded at 500 MHz, 25 °C.

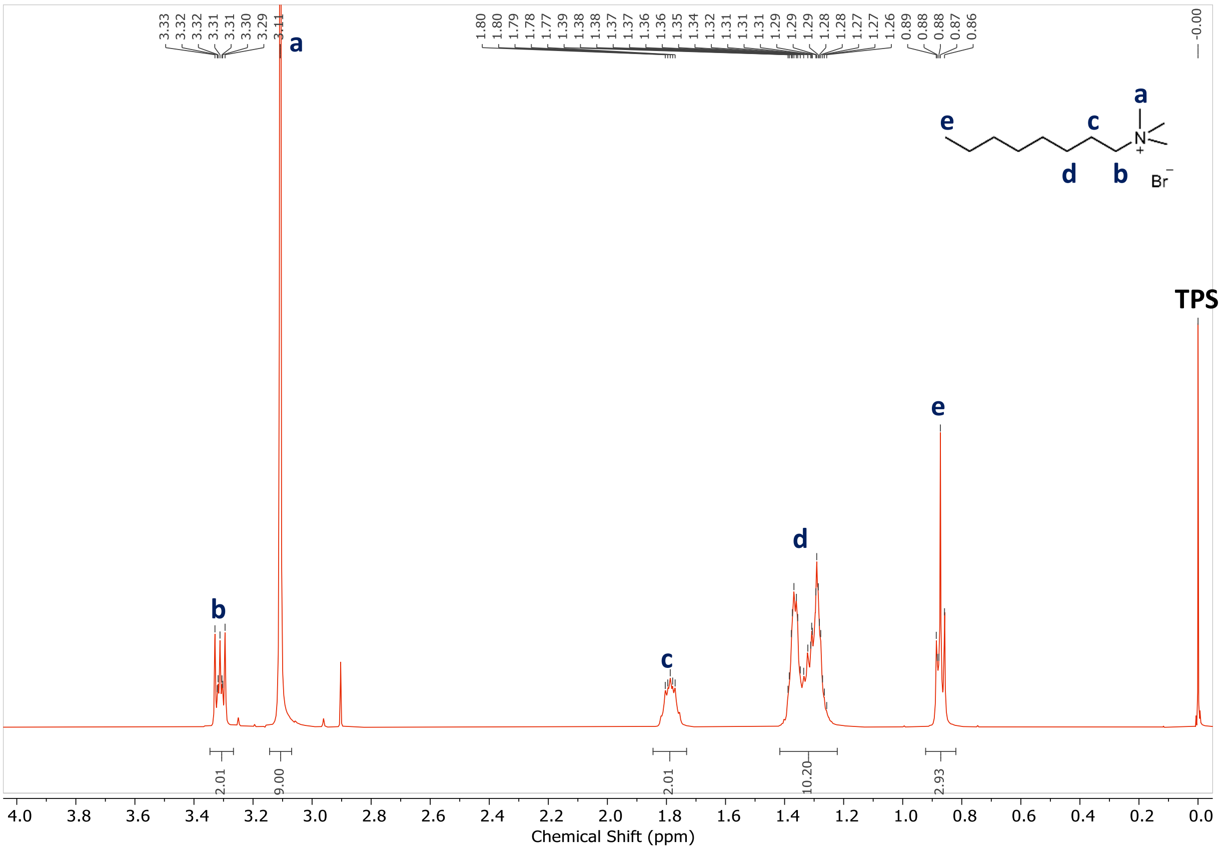

Figure S11: ^{1}H NMR spectrum after 24 h of well mixed [BTA]:[OTAB] (16:101 mM) in D_2O recorded at 500 MHz, 25 °C.

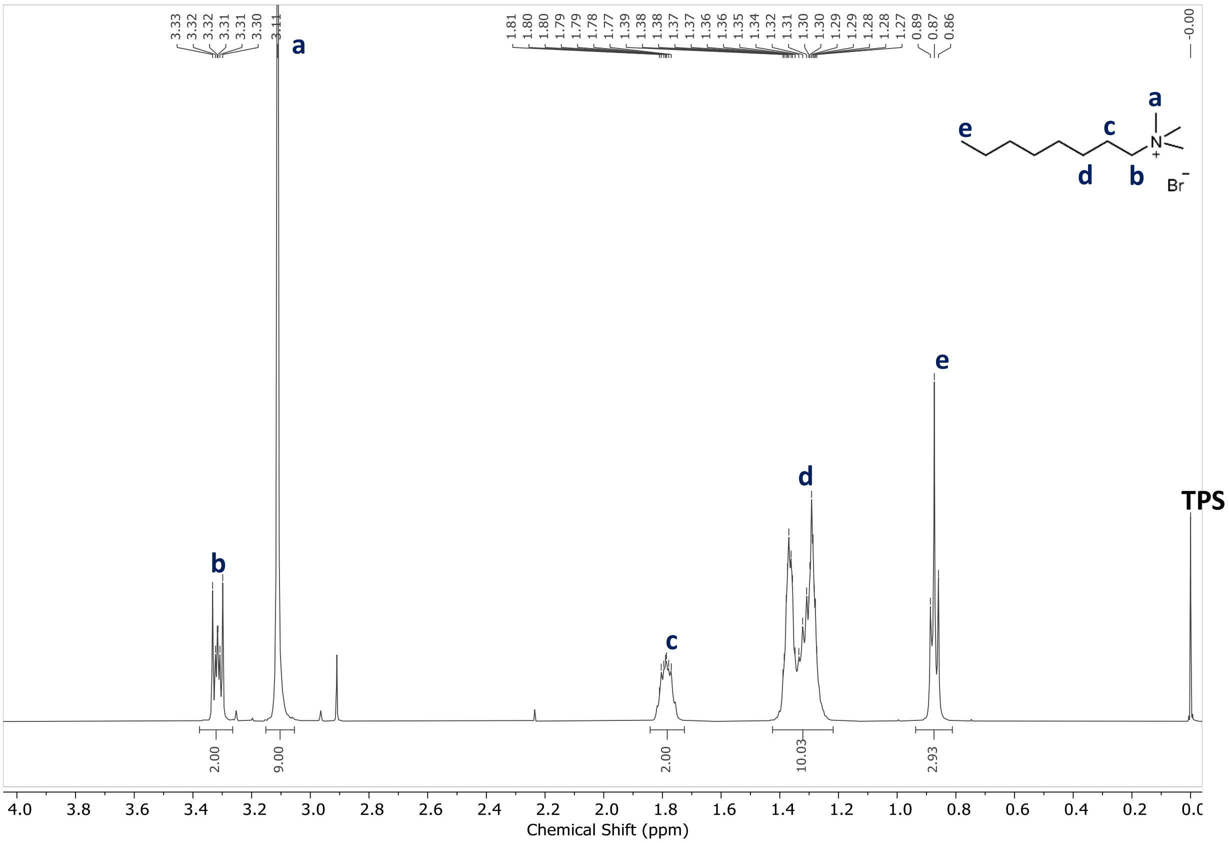

Figure S12: ^{1}H NMR spectrum of OTAB (202 mM) in D_2O recorded at 500 MHz, 25 °C.

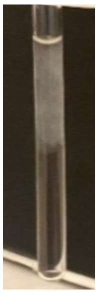

Figure S13: Picture of phase separation in NMR tube, directly after dilution to [BTA]:[OTAB] (16:101 mM) in D_2O.

Table S1: Integrals of BTA in ^{1}H NMR spectrum with decrease ratio upon transition from micelle to intermediate state, where decrese ratio = $\frac{\text{integral}_{micelle} - \text{integral}_{intermediate}}{\text{integral}_{micelle}}$.

	BTA_{EG^4}	$BTA_{N\text{-}CH}$	$BTA_{\text{aliphatic next to EG}^4}$	$BTA_{\text{aliphatic next to core}}$
Micelle [BTA]:[OTAB] 31:202 mM	6.98	0.87	1.19	5.41
Intermediate [BTA]:[OTAB] 16:101 mM	5.93	0.76	0.87	3.37
**Decrease ratio**	**0.150**	**0.126**	**0.269**	**0.377**

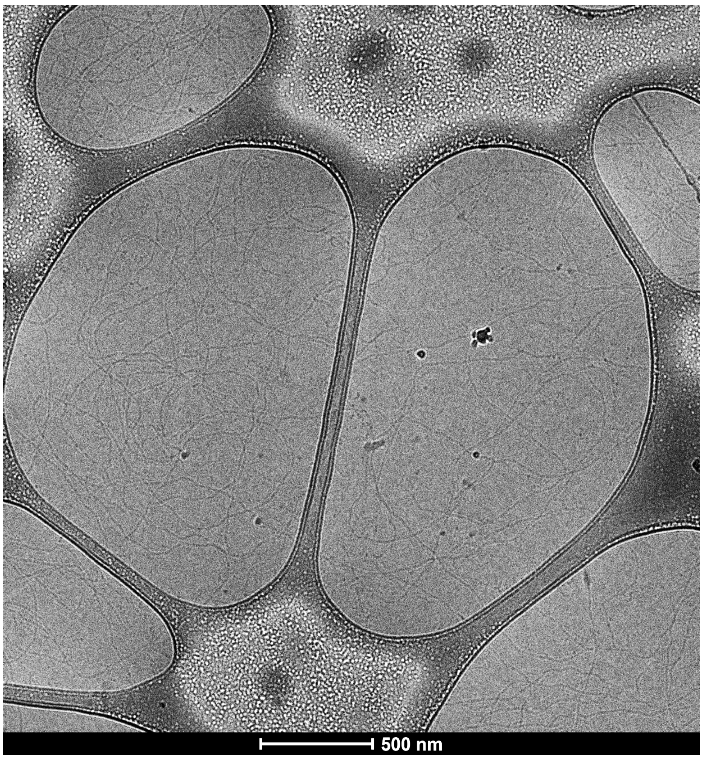

Figure S14: Cryo-TEM images of BTA-OTAB hydrogel (see *Sample preparation method VI*), showing 3D network entangled by micrometer long 1D fibers. The black spherical particles are ice-crystals. Scale bar = 500 nm.

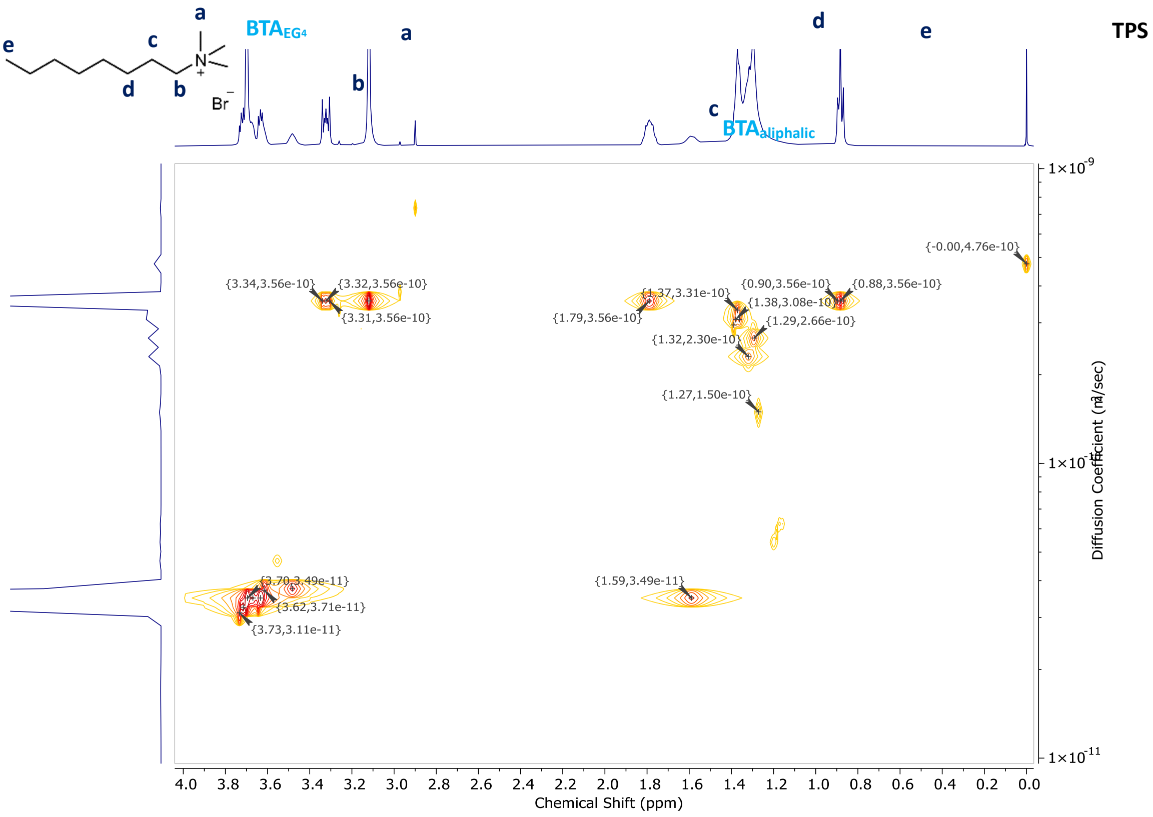

Figure S15: DOSY spectrum of [BTA]:[OTAB] (31:202 mM) in D_2O recorded at 500 MHz, 25 °C.

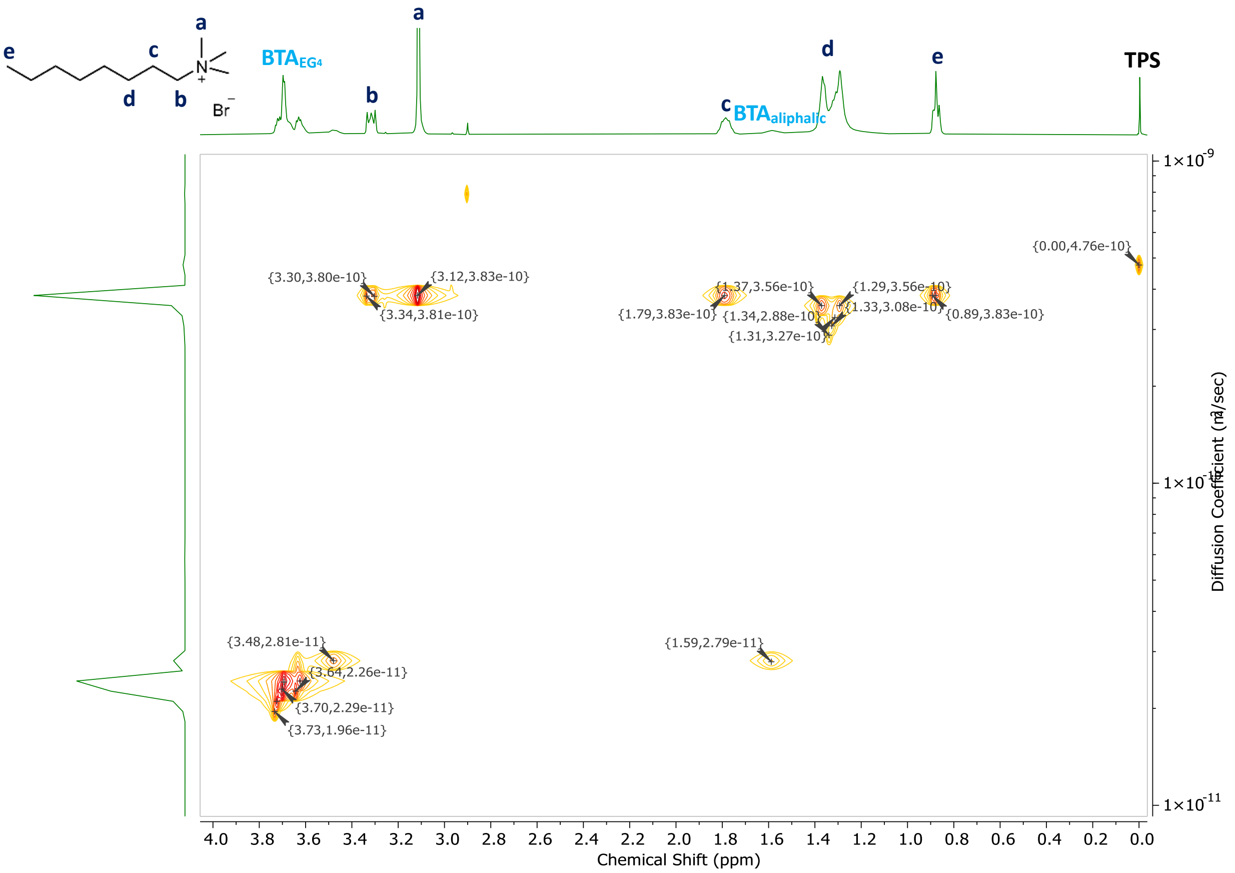

Figure S16: DOSY spectrum of [BTA]:[OTAB] (16:101 mM) directly after dilutions in D_2O recorded at 500 MHz, 25 °C.

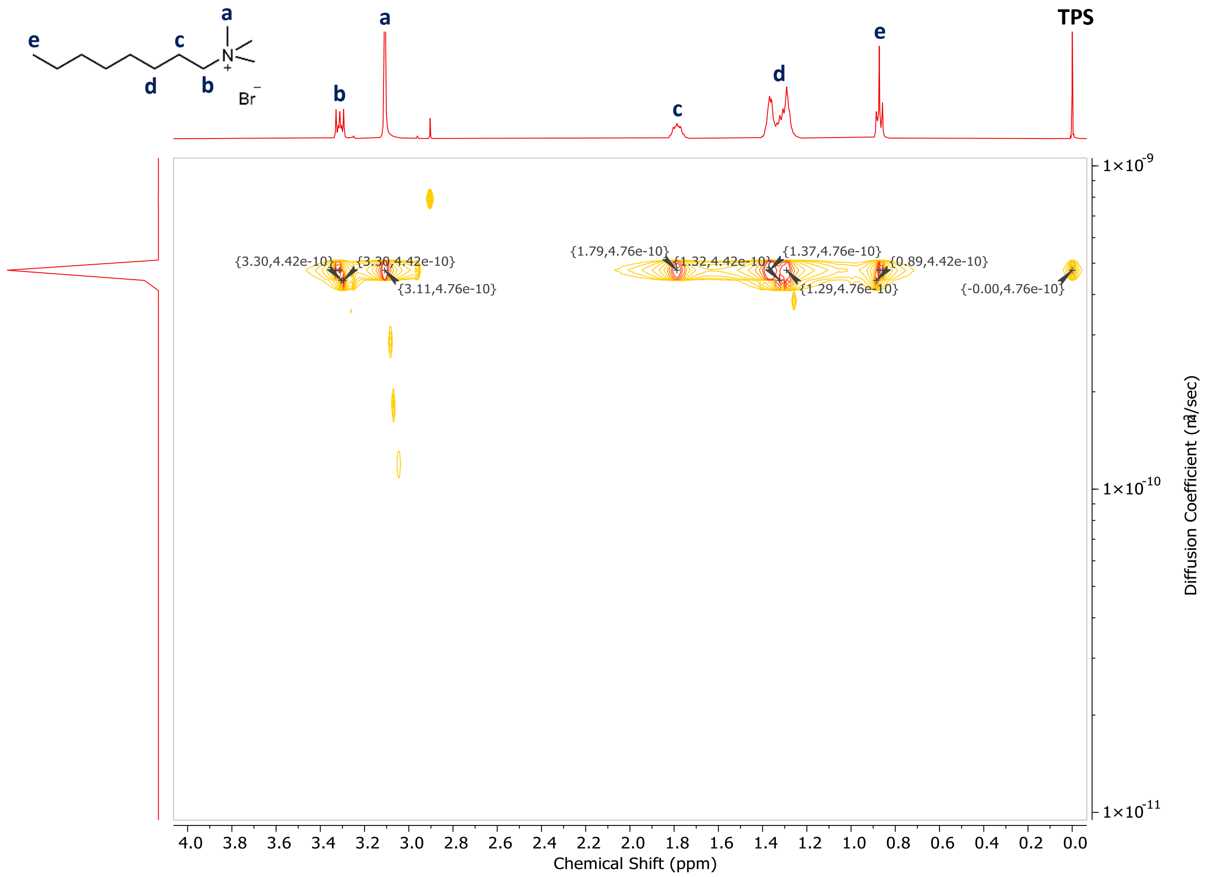

Figure S17: DOSY spectrum after 24 h of well mixed [BTA]:[OTAB] (16:101 mM) in D_2O recorded at 500 MHz, 25 °C.

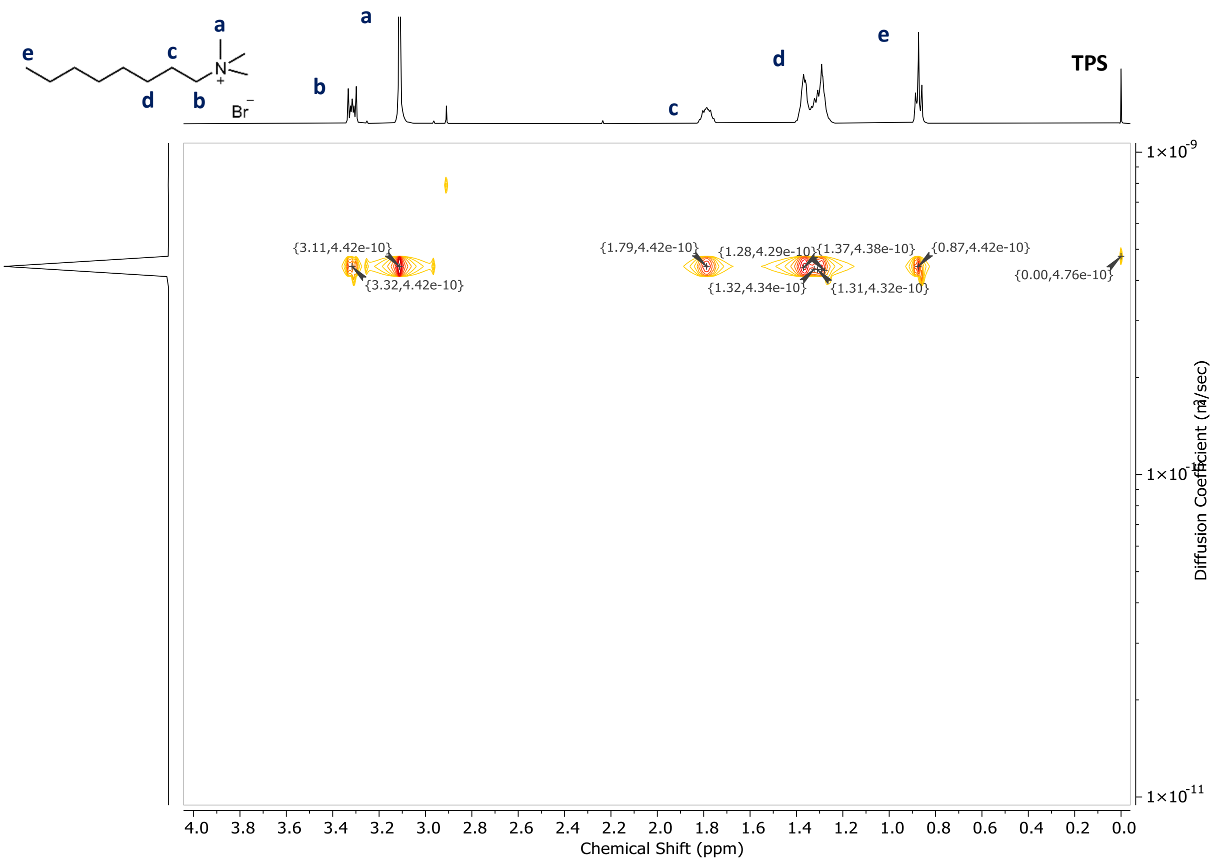

Figure S18: DOSY NMR spectrum OTAB (202 mM) in D_2O recorded at 500 MHz, 25 °C.

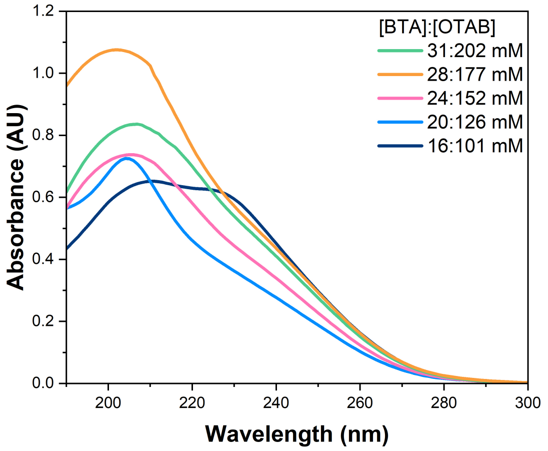

Figure S19: UV-vis spectrum of dilution of BTA-OTAB. UV-vis spectrum at different concentration of [BTA]:[OTAB].

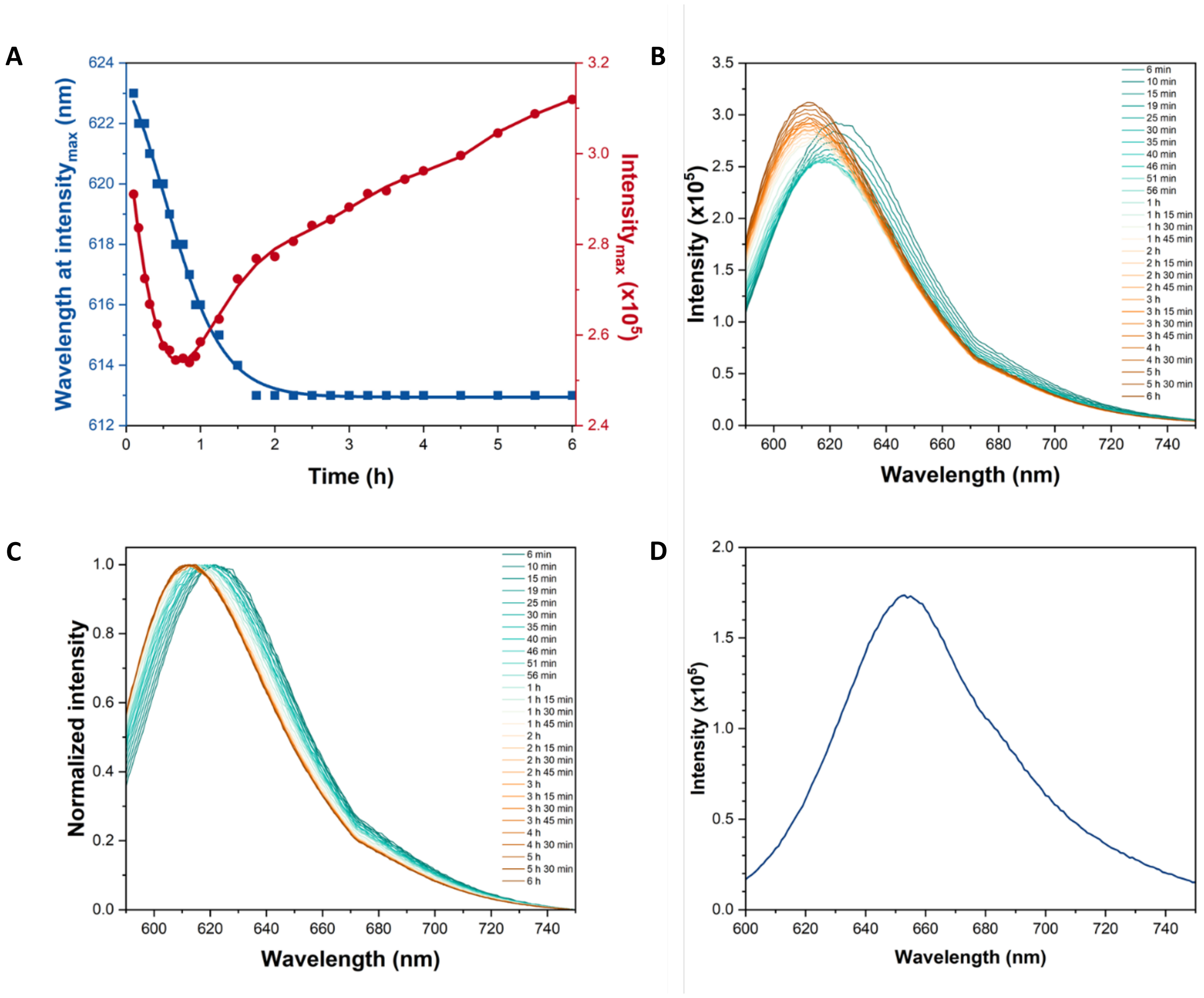

Figure S20: Time-dependent fluorescent emission of 20 µM Nile red in 2 x dilution of 31:202 mM [BTA]:[OTAB] solution of micelles. (A) Dilution results in supramolecular polymerization of which the wavelength and intensity of the maximum is monitored over 6 hours, showing shift of λ_{max} = 623 nm to 613 nm. (B) Full spectrum and (C) corresponding normalized spectrum. (D) control of 20 µM Nile red in MQ H_2O. (sample preparation II)

Note: Remarkably, the emission intensity decreased and then increased along with the blue shift, indicating the micelle to fiber (sol to gel) transition through an intermediate droplet state.[5]

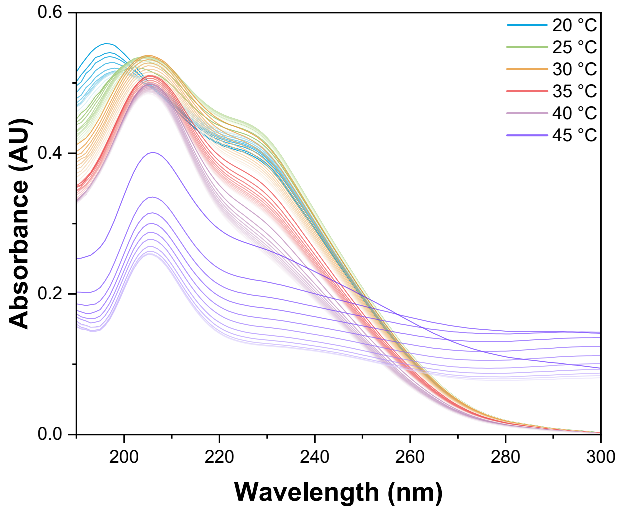

Figure S21: Temperature-dependent UV-vis spectrum of 16 mM BTA and 202 mM OTAB, mixed at rt. At each temperature, every min a spectrum was recorded for 10 min.

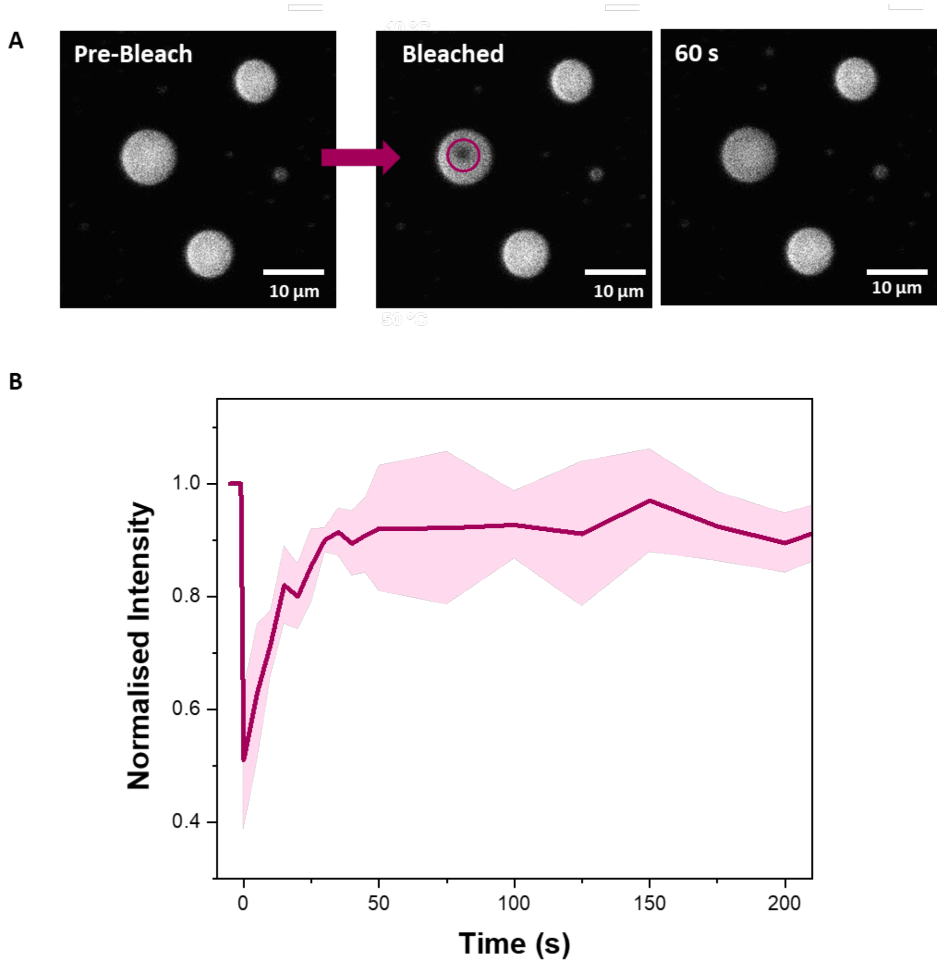

Figure S22: (A) CLSM images during FRAP at 50 °C. (B) Corresponding normalized FRAP kinetics. Scale bar = 10 µm.

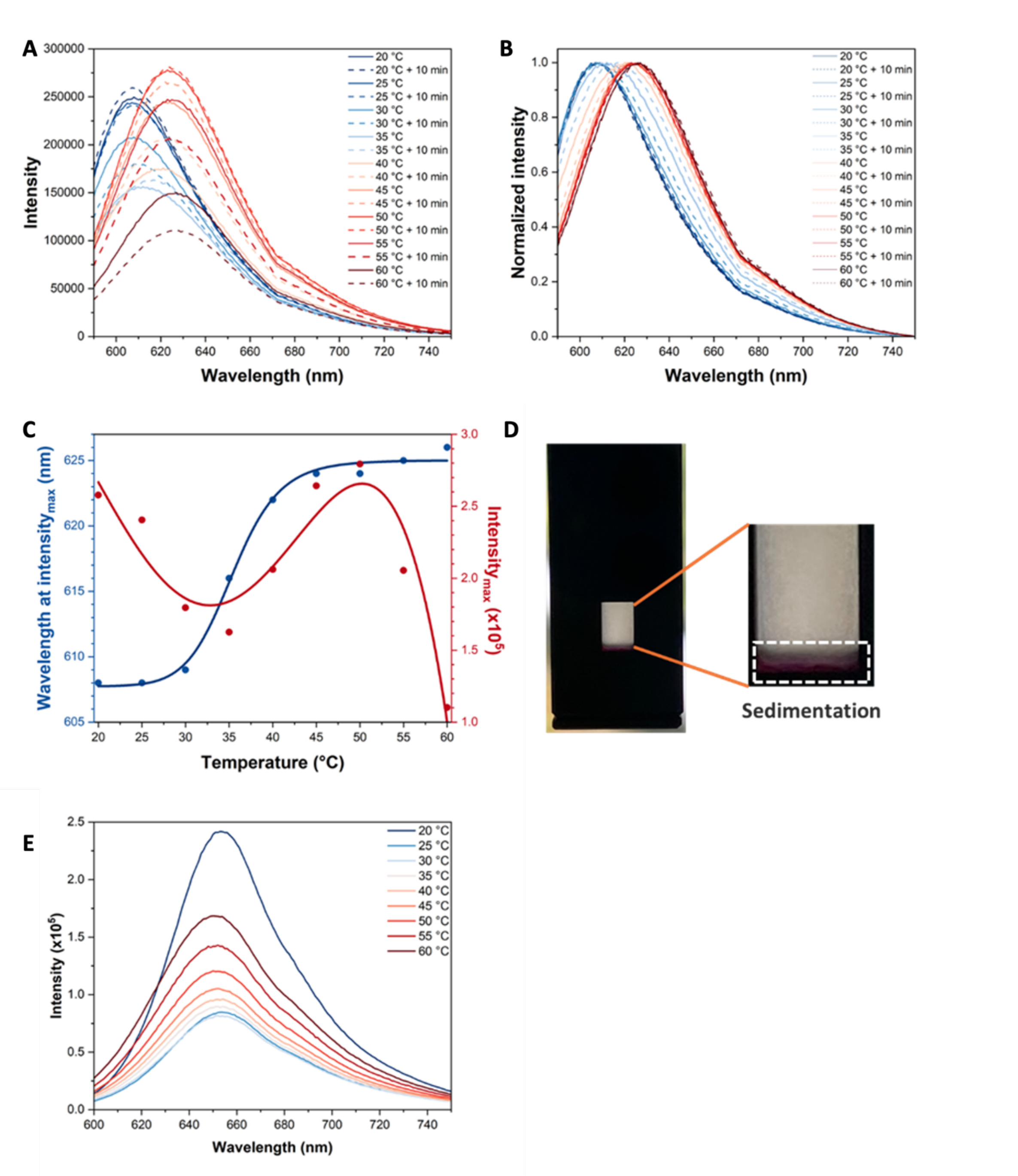

Figure S23: Temperature-dependent fluorescent emission of 20 µM Nile red in [BTA]:[OTAB] = 16:101 mM mixed at 22 °C. [BTA] = 16 mM is mixed with [OTAB] = 101 mM and Nile red = 20 µM at room temperature which is monitored from 20 °C to 60 °C, showing a shift of λ_{max} from 626 nm to 608 nm with (B) corresponding normalized spectra. (C) Maximum wavelength and intensity of temperature-dependent fluorescent emission assay. (D) Image of cuvette after measurement, showing sedimentation of condensed phase with Nile red. (E) Blue shift in emission spectrum λ_{max} from 653 nm to 650 nm over monitored from 20 °C to 60 °C in control of Nile red in MQ H_2O.

Note: The decrease in intensity of Nile red emission from 50 °C, is caused by merging of the liquid droplets. Here, a larger condensed phase is formed which sediments to the bottom of the cuvette.

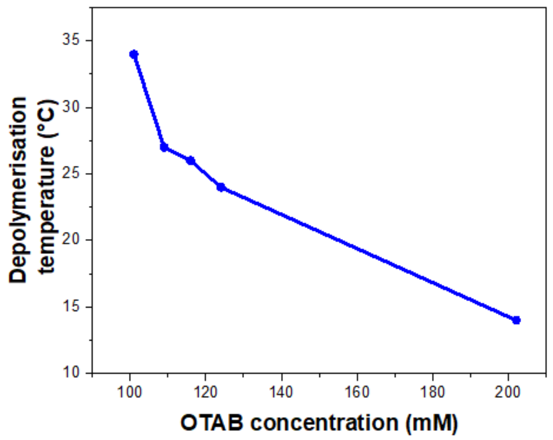

Figure S24: Variation of depolymerisation temperature with increasing OTAB concentration. [BTA] = 16 mM

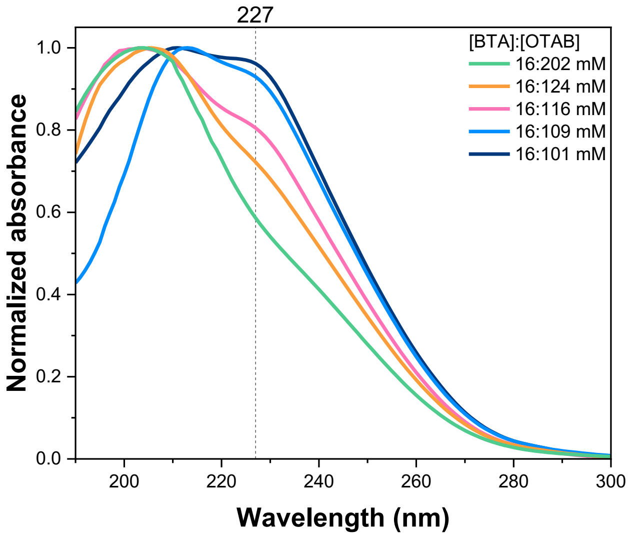

Figure S25: Normalized UV-vis spectrum of [BTA]:[OTAB] with varying OTAB concentrations, where [BTA] = 16 mM.

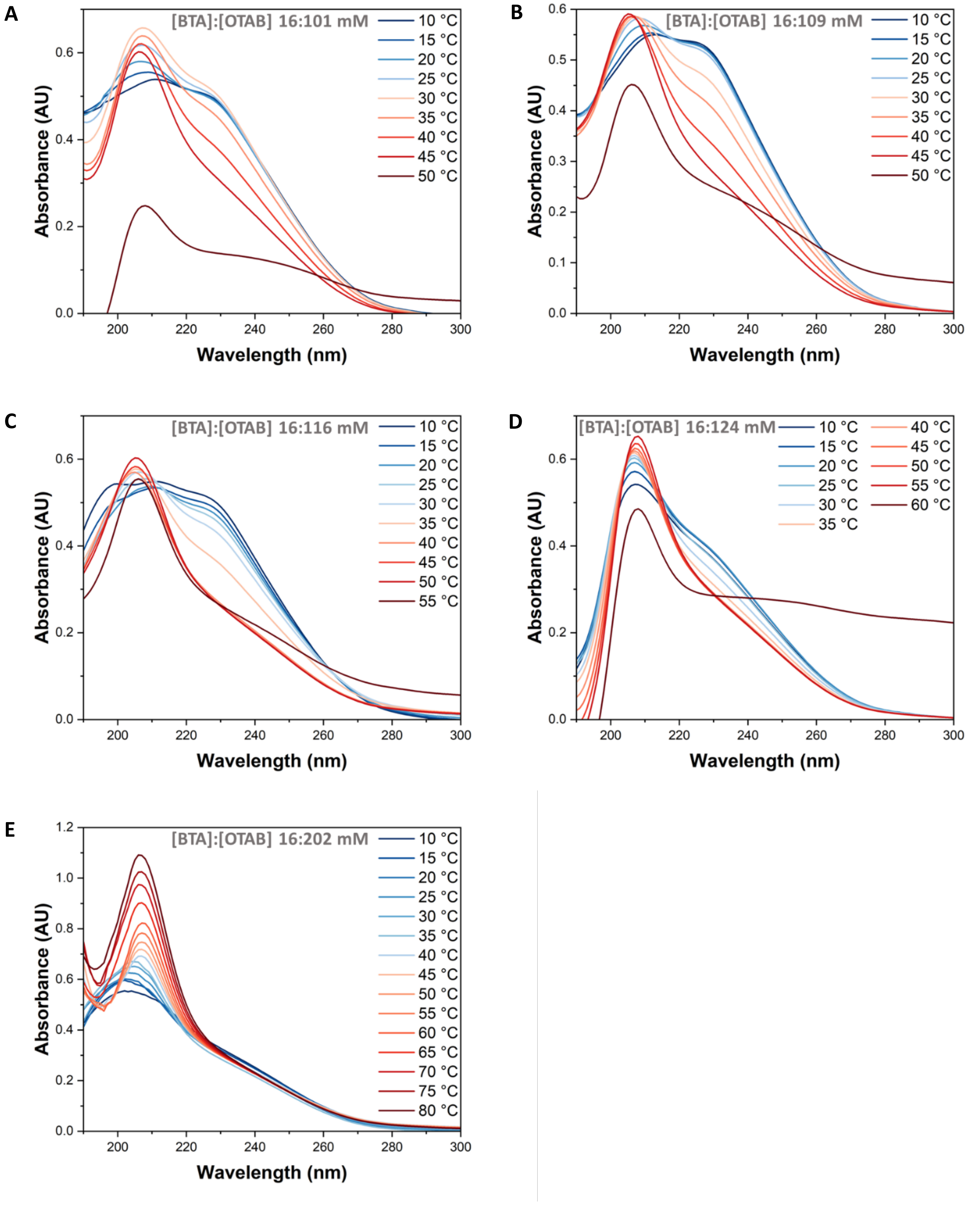

Figure S26: Temperature-dependent UV-vis spectra of several [BTA]:[OTAB] ratios. [BTA]:[OTAB] (A) 16:101 mM, (B) 16:109 mM, (C) 16:116 mM, (D) 16:124 mM and (E) 16:202 mM.

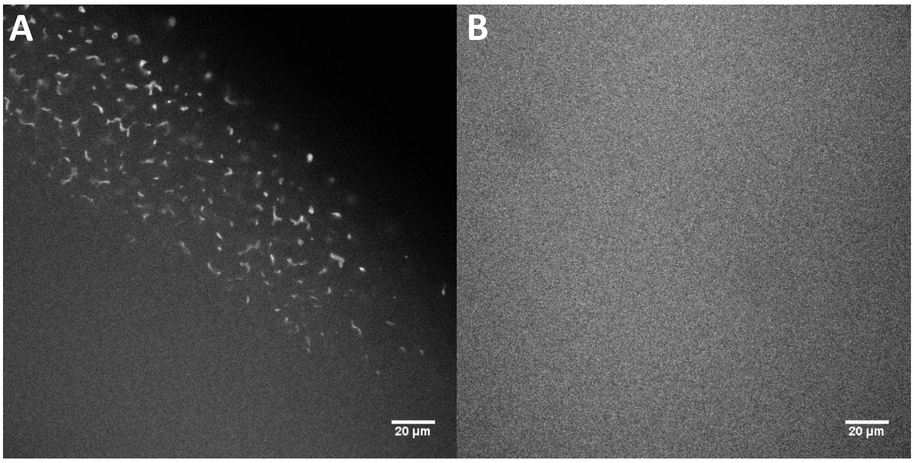

Figure S27: CLSM of BTA-OTAB assembly in presence of methanol. CLSM image of gradient of [BTA]:[OTAB] (31:202 mM) to (A) 5%, or (B) 8% methanol in MQ H_2O at 22 °C.

3. References

(1) Leenders, C. M. A.; Albertazzi, L.; Mes, T.; Koenigs, M. M. E.; Palmans, A. R. A.; Meijer, E. W. Supramolecular Polymerization in Water Harnessing Both Hydrophobic Effects and Hydrogen Bond Formation. *Chemical Communications* **2013**, *49* (19), 1963. https://doi.org/10.1039/c3cc38949a.

(2) Albertazzi, L.; van der Zwaag, D.; Leenders, C. M. A.; Fitzner, R.; van der Hofstad, R. W.; Meijer, E. W. Probing Exchange Pathways in One-Dimensional Aggregates with Super-Resolution Microscopy. *Science (1979)* **2014**, *344* (6183), 491–495. https://doi.org/10.1126/science.1250945.

(3) Fu, H.; Huang, J.; van der Tol, J. J. B.; Su, L.; Wang, Y.; Dey, S.; Zijlstra, P.; Fytas, G.; Vantomme, G.; Dankers, P. Y. W.; Meijer, E. W. Supramolecular Polymers Form Tactoids through Liquid–Liquid Phase Separation. *Nature* **2024**, *626* (8001), 1011–1018. https://doi.org/10.1038/s41586-024-07034-7.

(4) Patra, S.; Chandrabhas, S.; Dhiman, S.; George, S. J. Controlled Supramolecular Polymerization via Bioinspired, Liquid–Liquid Phase Separation of Monomers. *J Am Chem Soc* **2024**, *146* (18), 12577–12586. https://doi.org/10.1021/jacs.4c01377.

(5) Swain, J.; Mishra, A. K. Nile Red Fluorescence for Quantitative Monitoring of Micropolarity and Microviscosity of Pluronic F127 in Aqueous Media. *Photochemical & Photobiological Sciences* **2016**, *15* (11), 1400–1407. https://doi.org/10.1039/c6pp00123h.
